# Occurrence and concentrations of organic micropollutants (OMPs) in highway stormwater: a comparative field study in Sweden

**DOI:** 10.1007/s11356-023-27623-9

**Published:** 2023-05-31

**Authors:** Ali Beryani, Kelsey Flanagan, Maria Viklander, Godecke-Tobias Blecken

**Affiliations:** Department of Civil, Environmental, and Natural Resources Engineering, Luleå University of Technology, 97187 Luleå, Sweden

**Keywords:** Road runoff, Quality monitoring, Monte-Carlo simulation, Uncertainty analysis, Censored data, Correlated parameters

## Abstract

This study details the occurrence and concentrations of organic micropollutants (OMPs) in stormwater collected from a highway bridge catchment in Sweden. The prioritized OMPs were bisphenol-A (BPA), eight alkylphenols, sixteen polycyclic aromatic hydrocarbons (PAHs), and four fractions of petroleum hydrocarbons (PHCs), along with other global parameters, namely, total organic carbon (TOC), total suspended solids (TSS), turbidity, and conductivity (EC). A Monte Carlo (MC) simulation was applied to estimate the event mean concentrations (*EMC*) of OMPs based on intra-event subsamples during eight rain events, and analyze the associated uncertainties. Assessing the occurrence of all OMPs in the catchment and comparing the *EMC* values with corresponding environmental quality standards (EQSs) revealed that BPA, octylphenol (OP), nonylphenol (NP), five carcinogenic and four non-carcinogenic PAHs, and C_16_-C_40_ fractions of PHCs can be problematic for freshwater. On the other hand, alkylphenol ethoxylates (OPnEO and NPnEO), six low molecule weight PAHs, and lighter fractions of PHCs (C_10_-C_16_) do not occur at levels that are expected to pose an environmental risk. Our data analysis revealed that turbidity has a strong correlation with PAHs, PHCs, and TSS; and TOC and EC highly associated with BPA concentrations. Furthermore, the *EMC* error analysis showed that high uncertainty in OMP data can influence the final interpretation of *EMC* values. As such, some of the challenges that were experienced in the presented research yielded suggestions for future monitoring programs to obtain more reliable data acquisition and analysis.

**Supplementary Information:**

The online version contains supplementary material available at 10.1007/s11356-023-27623-9.

## Introduction 

Stormwater runoff is a primary source of toxic organic substances in urban surface waters and near-shore sediments (Barbosa et al. 2012; Launay et al. 2016; Luo et al. 2014). Among different types of urban catchments, roads represent one of the most important sources of certain carcinogenic trace organic compounds, such as polycyclic aromatic hydrocarbons (PAHs) (Diblasi et al. 2009; Wicke et al. 2021). However, traffic-related activities can also release many other types of organic pollutants which may be resistant to environmental degradation, demonstrate bioaccumulation tendencies, and potentially cause long-term detrimental effects on aquatic life (Diblasi et al. 2009; Markiewicz et al. 2017). Recent studies by Wicke et al. (2021) and Gasperi et al. (2022) showed that five and seven organic micropollutants (OMPs), respectively, including phthalates, alkylphenols, and PAHs, can be found in stormwater road runoff at levels that exceed European environmental quality standards (EQS) for surface waters. Mutzner et al. (2022) also revealed that seven traffic-related organic substances (all PAHs) are among the top ten top micropollutants that pose an environmental risk in urban wet-weather flows, which occurred in > 92% of stormwater sampling sites around the world. Although OMP concentrations in receiving bodies are often expected to be lower than acute toxic levels of concern (due to dilution), OMPs can nevertheless cause long-term chronic negative effects, either directly or after interacting with other substances (Gerbersdorf et al. 2015; Rehrl 2019). For instance, a recent eco-toxicological field study confirmed that hydrophilic trace organic substances found in stormwater runoff exert adverse acute and chronic effects on some aquatic species in the receiving surface water (Spahr et al. 2020).

Nevertheless, there are some clear differences in how certain runoff pollutants are represented in the highway stormwater quality database. Previous studies have extensively characterized contamination arising from trace metals, nutrients, oil and grease, COD, and TSS, among others. However, despite the importance of the OMPs described above, there is still limited information about the levels at which these compounds are found in road runoff (Gasperi et al. 2022; Mutzner et al. 2022). Research into this area has increased on a global level in recent years, but prior studies have mainly focused on polycyclic aromatic hydrocarbons (PAHs) while largely ignoring emerging substances, such as phenolic substances, despite their potential environmental risks. Given the importance of OMPs in road runoff, the present work aims to investigate the occurrence of poorly studied organic substances (bisphenol-A (BPA), 4-t-octylphenol (OP), nonylphenol (NP), and OP- and NP-ethoxylates) as well as common OMPs (sixteen PAHs and four fractions of petroleum hydrocarbons (PHCs)). More in-depth knowledge of OMP sources and concentrations can signal the risk that these compounds pose for receiving waters and, subsequently, contribute to the development of road runoff quality management strategies both in the forms of source control and treatment in specific catchments.

Another important aspect to study is the associations between different stormwater organic contaminants (especially new emerging OMPs), as well as how the levels of OMPs are associated with conventional water quality parameters (e.g., suspended solids, organic carbons, turbidity, and conductivity). The associations may reveal novel information about identifying potential conventional indicator parameters that can be used to follow the trends of the studied OMPs with similar environmental fates and transport behaviors (Mutzner et al. 2020), although establishing such parameters using correlations requires extensive investigations and would be site-specific. For example, one application can be that the concentrations of specific OMPs are continuously evaluated at high time-resolution using a relatively small set of constituent parameters in certain stormwater monitoring programs on this site. This complementary information can eventually be useful for runoff pollutant prioritization, source identification, reducing analytical costs, improving economic water quality monitoring programs, monitoring the effectiveness of best management practices (BMPs), as well as determining further treatment possibilities.

The first objective of this study was to monitor the occurrence and concentration levels of target OMPs in road runoff from a highway bridge catchment in Sweden. Regarding this objective, the event mean concentrations (EMCs) were compared with highway/road runoff characteristics reported in the literature and surface water quality objectives (WQO) to determine the possible risks of the detected OMPs. The second objective was to identify the association of conventional water quality parameters with the selected OMPs for future monitoring purposes at this specific site.

## Methodology

### Study site

The field study was carried out in Sundsvall, Sweden, which has a continental subarctic climate (Dfc) and cool summers. The impervious catchment area comprises 4.7 ha, of which 1.9 ha are included in a highway bridge (E4) with an average traffic load of 13,000 vehicles/day (Fig. S1). The rest of the area consists of a highway exit, an acceleration ramp entering the highway, main roads associated with two roundabout, and sidewalk paths.

Sundsvall E4 bridge was built and opened to public in 2014. The pavement surface asphalt was constructed using a polymer-modified bitumen (PMB20) product (Endura F2), especially designed for bridge applications, for which the wearing course of the pavement is made of polymer modified stone mastic asphalt (ABS 11) (Lu et al. 2016; Nynas.com 2018). The PMB product has excellent aging resistance and high elasticity and exceptional flexibility, resulting in sustaining large strains/stresses at a wide range of temperatures (Lu et al. 2016). The pavement surface was in a good condition. According to the Swedish Pavement Management System (PMSv3), some rutting tests on another part of E4 highway pavement under the similar conditions (i.e., same asphalt mixture, climate region, and traffic load as for this study) showed that the rutting development for the PMB20 is very insignificant over 8 years (Lu et al. 2016), meaning that the damage to the asphalt concrete is expected to be very low at the time of experiment, but the residual pavement wear particles might still be a source of organic pollutants in the runoff.

### Sampling procedure and strategies

The number of events needed to reliably estimate mean OMP concentrations in the stormwater outlets of a study site depends on local conditions, along with the characteristics and variability of each micropollutant. A metastudy on stormwater outlets across 55 sites found the median number of selected events to be six (with Q10 and Q90 quantiles of 1 and 25 events) (Mutzner et al. 2022). Also, studies on micropollutants typically sample between 1 and 12 events (Spahr et al., 2020). In the present study, we investigated stormwater quality for eight rain events (referred to as rains A to H) between September 2020 and September 2021 (Table 1). As shown in Table 1, the sampling events only covered the warmer seasons, and none of them was performed during winter conditions or snow-melting periods, so the effect of de-icing salt and pollution accumulation in snow deposits were not investigated in this study.

**Table 1:** Rain event characteristics, sampling, and flow volume information

Rain event characteristics							Total volume conveyed and the percentage sampled	
Rain event	Sampling date (2020 − 21)	Depth (mm)	Duration (h)	ADP^¥^ (day)	Mean intensity or *I*_mean_ (mm/h)	Peak intensity or *I*_peak_ (mm/h)	Tot. volume passed* (m^3^)	Tot. volume sampled (%)
A	15 Sept	7.3	14.3	3	1.5	5.5	257	100%
B	21 Oct	4.6	13.8	0.8	0.7	2.4	195	100%
C	17 May	7.8^§^	3.25^§^	1.7^§^	2.6 ^§^	12.0 ^§^	373	100%
D	30 Jun	3.8	18.6	6.7	2.3	8.6	128	83%^$^
E	11 Jul	8.3	13.7	10.5	9.4	32.7	280	100%
F	20 Aug	32.4	40.9	1	3.7	21.2	2353	79%^#^
G	25 Aug	7.9	46.7	6.3	1.4	4.2	303	96%
H	24 Sep	17.8	24.4	10.4	1.7	4.8	932	59%

^¥^Antecedent dry period (events with total precipitation < 1 mm were excluded)

^*^Estimated by the number and volume of pulses (~ 23.3 m3/pulse) received from the GPT discharge valve

^$^The first portion of the inflow was not sampled

^§^Reported by the nearest station to the catchment (1500 m away from the rain gauge at the facility)

^#^21% of inflow was not sampled during the last quarter because of at least one of the following reasons: bypassed overflow (16%); GPT discharge valve stayed open due to a very high inflow (no new signal to the SW and GPT sampler was sent in this mode); or the sampling program had ended (final 5%)

All of the samples were collected from the end of a storm sewer pipe (length: ~ 100 m; diameter: 800 mm; slope: 0.5%) which was downstream of the highway catchment. Rainfall data were recorded using a tipping bucket rain gauge (ISCO 674; Teledyne ISCO, Lincoln, NE) that was situated directly beside the highway. An ISCO-6712 automatic sampler (Teledyne ISCO) was used to collect volume proportional samples during the rain events.

Flow volume at the sampling point was measured by counting the signals sent by discharge valves of a gross pollutant trap chamber (GPT chamber) located downstream of the pipe outlet to the sampler. Every 23.3 m^3^, a balloon-type floating switch in the GPT chamber sent a signal to the GPT discharge valves to open/close and, at the same time, triggered the automatic sampler to take a subsample. Based on forecasted precipitation, the sampler was programmed to collect a maximum of eight volume-proportional subsamples. Table 1 summarizes the precipitation characteristics and flow information at the sampling point for all events. For some rain events, we could not cover the entire runoff volume due to practical limitations such as rain depth, duration uncertainties, and time constraints in delivering the samples.

The sampler was equipped with 24 Teflon bags (lay flat PFA bag, Welch Fluorocarbon, Dover, NH), with every three bags represented one subsample. The Teflon bags were washed thoroughly with tap water before sampling. After the sampling procedure, the samples were delivered to the laboratory within 1 day. In case of delivery time lag (e.g., weekends), all OMP samples were stored in a dark and cool place (1–4 °C), except for TOC samples, which were stored in a freezer (< − 15 °C).

### Water quality analysis

All of the samples are analyzed for the OMPs listed in Table 2 and global parameters, including total organic carbon (TOC), total suspended solids (TSS), turbidity, conductivity, pH, DO, and temperature. OMPs and TOC were analyzed by the ALS Czech Republic Laboratory (Prague, Czechia; accredited by the Czech Accreditation Institute) and TSS by ALS Scandinavia AB (Luleå, Sweden; holding Swedac accreditation). Gas chromatography with MS or MS/MS detection was used for analyzing the OMPs present in samples. According to the lab reports, the chemical analyses included matrix interference between alkylphenol substances, which occasionally affected the limits of quantification (LoQ) (Table 2). In addition, other global parameters, including turbidity, conductivity, pH, and temperature, were measured for each subsample. Further information about the analytical methods and equipment are provided in Table S1.

**Table 2:** List of organic micropollutants (OMPs) analyzed in the stormwater samples

	Organic substances, abbreviations, and limits of quantification (LoQs in µg/L)
Phenolic substances	*Bisphenol-A*: **BPA** (0.05); *Nonylphenol, mixture of isomers*: **NP** (0.1–1.35*); *Nonylphenol monoethoxylate*: **NP1EO** (0.1–0.3*); *Nonylphenol diethoxylate*: **NP2EO** (0.1–2.54*); *Nonylphenol triethoxylate*: **NP3EO** (0.1–3.12*); *4-tert-Octylphenol*: **OP** (0.01–0.25*); *Octylphenol monoethoxylate*: **OP1EO** (0.01–0.03*); *Octylphenol diethoxylate*: **OP2EO** (0.01–0.02*); *Octylphenol triethoxylate*: **NP3EO** (0.01–0.033*)
Polycyclic aromatic hydrocarbons (PAHs)	Substances: *Naphthalene*: **Nap** (0.03); *Acenaphthylene*: **Acyl** (0.01); *Acenaphthene*: **Acen** (0.01); *Fluorene*: **Flu** (0.01); *Phenanthrene*: **Phen** (0.02); *Anthracene*: **Anth** (0.01); *Fluoranthene*: **Flth** (0.01); *Pyrene*: **Pyr** (0.01); *Benz(a)anthracene*: **BaA** (0.01); *Chrysene*: **Chry** (0.01); *Benzo(b)fluoranthene*: **BbF** (0.01); *Benzo(k)fluoranthene*: **BkF** (0.01); *Benzo(a)pyrene*: **BaP** (0.01); *Dibenz(a.h)anthracene*: **DahA** (0.01); *Benzo(g.h.i)perylene*: **Bper** (0.01); *Indeno(1.2.3-cd)pyrene*: **InP** (0.01) Fractions: *Sum of all*: **∑16 PAH**; *carcinogenics*: **∑Car PAH** (Nap, BaA, Chry, BbF, BkF, BaP, BahA, and Bper); *Non-carcinogenic*: **∑nonCar PAH** (Acyl, Acen, Flu, Phen, Anth, Flth, Pyr, and InP); *low-weight molecules:* **∑LMW PAH** (Nap, Acyl, Acen, Flu, Phen, and Anth); *medium-weight molecules:* **∑MMW PAH** (Flth and Pyr); and *high-weight molecules*: **∑HMW PAH** (BaA, Chry, BbF, BkF, BaP, DahA, Bper, and InP)
Petroleum hydrocarbons (PHCs)	Fractions: *Total PHCs or* **C**_**10**_**-C**_**40**_ (50); **C**_**10**_**-C**_**12**_ (5); **C**_**12**_**-C**_**16**_ (5); **C**_**16**_**-C**_**35**_ (30); **C**_**35**_**-C**_**40**_ (10)

*The upper limit of the range includes a shift in the limit of quantification (LoQ) due to matrix interferences during chemical analysis

Batch leaching tests were conducted to investigate whether OMPs could have leached from the sampling equipment (the Teflon bags and sampler suction line). The suction line has two parts: a 1.3-m silicon rubber hose at the sampler’s pumping head and a 13.5-m Teflon-lined LDPE tube attached to a stainless-steel strainer at the other end. First, tap water was pumped through the sampler (through the tubes described above) and poured into both a new and a used Teflon bag. The water was then kept in the Teflon bags for 24 h. Next, the water was analyzed for OMPs of concern. Comparisons of the concentrations of certain analytes in the reference batch water and water that had been kept in the Teflon bags for 24 h revealed that the sampling equipment did not leach phenolic substances. However, TOC concentrations increased from below LoQ (0.5 mg/L) to 0.52–1.36 mg/L (less than 10% of the mean TOC value measured in this study). Field pretests on grab samples also showed that the sampling equipment did not become contaminated by either PAHs or TPHs. Nevertheless, the Teflon bags were replaced after the first 6 to 7 events. Moreover, each bag was assigned to the same position in an identical sampler to avoid cross-contamination throughout the experiment.

### Data analysis

#### EMC

The event mean concentration (*EMC*) for each OMP was calculated based on subsample concentrations and flow data. The *EMC* represents the total pollutant mass (*M*_*T*_) conveyed by the total runoff volume (*V*_*T*_) during the entire sampling period of each rain event (Eq. (1)). However, for events in which the subsamples do not cover the whole stormwater volume, the *EMC* represents a partial mean concentration for the sampled portion of the rain event. A majority of the instances in which the subsamples do not cover the entire rain event were missing the end of the event (Table 1), with the exception of rain event D, for which the first 17% of stormwater was not sampled. Therefore, a decision was made to use suggestions from Furuta et al. (2022) to more accurately estimate total *EMC* by attributing the missing volume to the volume of the last subsample under the assumption that the concentrations between the missing volume and final subsample are equivalent. Similarly, the missing period of rain event D was attributed to the first subsample in the total *EMC* calculation.

1 $$EMC=\frac{{M}_{T}}{{V}_{T}}=\frac{\sum {m}_{i}}{\sum {v}_{i}}=\frac{{\sum }_{i=1}^{n}{c}_{i}{v}_{i}}{{\sum }_{i=1}^{n}{v}_{i}}$$

where *EMC* is the stormwater event mean concentration; *n* is the number of subsamples; *m*_*i*_ is the mass of pollutant conveyed in the period during which the *i*th subsample was taken; *c*_*i*_ and *v*_*i*_ represent the concentration measured in the *i*th subsample, and the corresponding volume of stormwater, respectively.

The calculated *EMC* values were briefly compared with the lowest predicted no-effect concentrations (PNEC) reported in the NORMAN Ecotoxicology Database and/or with annual average Environmental Quality Standards (AA-EQS) prioritized by the European Union Water Framework Directive (2013/39/EU); both serve as water quality reference points for freshwater.

### EMC estimation and uncertainty propagation using a Monte-Carlo method

A Monte-Carlo (MC) method was applied to estimate the *EMC*, along with the uncertainty associated with each *EMC* value (Bertrand-Krajewski et al. 2021). MC uncertainty propagation is easy to interpret and can be used to assign various uncertainty distributions to different data types (Albert 2020). One source of uncertainty in input variables is the analytical measurement uncertainties (± *δ*_*i*_) of subsample concentrations (*c*_*i*_). The laboratory reported the *δ*_*i*_ values as extended uncertainties (JCGM 100,2008) with a coverage factor of two (covering a ~ 95% confidence level) only for quantified substances with concentrations above the LoQ. In this case, the uncertainty distribution for each *c*_*i*_ was assumed to be *normal* for detected substances. When a substance was not quantified in a subsample (i.e., the concentration was left-censored), the uncertainty distribution was assumed to be a uniform distribution between zero and the LoQ, as shown in Eq. (2). The volume that passed in the first subsamples (*v*_*1*_) was also assumed to have a *uniform* distribution due to the possible presence of runoff in the GPT chamber before the start of the event. We expected a negative uncertainty in the 21 m^3^ of water (90% of the GPT chamber’s discharging volume) that passed during the first subsample period (*v*_1_). For the subsequent subsamples, we assumed that the uncertainty of *v*_*i*_ values calculated by the number of valve opening pulses and the known GPT chamber’s discharging volume (23.3 m^3^) would be insignificant.

2 $$\left\{\begin{array}{l}C_i\sim N\;(\mu=c_i,\sigma=\delta_i/2)\;if\;C_i\;detected\\C_i\sim U\;(min=0,max=LoQ)\;if\;C_i\;censored\\V_1\sim U\left(min=v_1-21^{(m^3)},\;max=v_1\right)\end{array}\right.$$

In the MC method, Eq. (1) was used to obtain the *EMC*. The first *EMC* value was calculated via random sampling from the specified uncertainty distributions of all *n* input values in Eq. (2) under the assumption that the uncertainties are independent. Then, this calculation procedure was repeated 10^5^ times with new drawings at each iteration. According to the rules of the MC method, the distribution generated by many random trials can directly indicate the best estimation for *EMC* and the associated distribution parameters (mean, standard deviation or SD, and confidence intervals or CI). The median of the trial *EMC* values was referred to as *EMC*_best_, while the 2.5 and 97.5% quantiles represent the lower (*Δ*_*l*_) and upper (*Δ*_*u*_) uncertainty levels, respectively, for *EMC* (*Δ*_*l*_ ≤ *EMC* ≤ *Δ*_*u*_ with CI 95%). The *SD* of the randomly-generated *EMC* values was also reported as a statistical parameter of the resulting distribution (with *µ* = *EMC*_best_). In the case that all of the subsample concentrations were censored, the *EMC* was reported as occurring between zero and the maximum LoQ observed.

The described MC method was applied twice to estimate the *EMC* values and analyze the uncertainties for six different fraction groups of PAHs (PAH-16Sum, PAH-Car, PAH-nonCar, PAH-LMW, PAH-MMW, and PAH-HMW). In the first step, concentration distributions for the fraction groups in each subsample were generated using single PAH substance data. Next, these distributions were used to estimate the *EMC*_best_ and uncertainty levels of the fraction groups.

#### Statistical and censored data analysis

All of the *EMC* value estimations, including uncertainty analysis and statistical tests, were carried out in R software (V4.1.3). In addition, the *NADA* (non-detects and data analysis for environmental data) package in R was used for the statistical analysis of *EMC* datasets which contained non-detects. After MC calculations, a non-exceedance probability (NEP) plot of the *EMCs* for all rain events was generated to evaluate *EMC* estimation error and statistical distribution among all events. In NEP plots, the *y*-axis represents the cumulative probability of the calculated *EMCs* (shown on the *x*-axis) using the Kaplan–Meier method, which is commonly applied for censored data analysis (Helsel 2010). *EnvStats* (V2.3.0), a comprehensive R package for environmental statistics, was used to calculate the cumulative probabilities through the Kaplan–Meier method. The calculated *EMCs* were briefly compared with the lowest predicted no-effect concentrations (PNEC) reported in the NORMAN Ecotoxicology Database and/or annual average Environmental Quality Standards (AA-EQS) prioritized by the European Union Water Framework Directive (2013/39/EU); both serve as water quality reference points for freshwater.

The correlations between all parameters, including the *EMC* of OMPs and global parameters and rain characteristics (rain depth, mean intensity or *I*_mean_, peak intensity or *I*_peak_, and antecedent dry period), were evaluated using non-parametric pairwise Spearman’s rank correlation or Kendall’s tau test with *N* = 8 (number of events). Given that most parameters over the rain events did not follow a normal distribution (according to a Shapiro–Wilk normality test), the first test (Spearman’s rank correlation) was used for the detected data sets. Therefore, the latter test was applied to determine correlations between two data sets of which at least one included left-censored data. The resulting correlation coefficients were then classified as very strong, strong, moderate, weak, and very weak (> 0.9, > 0.7, > 0.3, > 0.1, and < 0.1, respectively, for Spearman’s rank correlation; > 0.7, > 0.5, > 0.2, > 0.1, and < 0.1, respectively, for Kendall’s tau test). The statistical significance of association was accepted if the *p*-value ≤ 0.05 in both tests (H_0_: There is no true correlation between the two parameters).

## Results and discussion

### The occurrence and concentrations of OMPs

**Fig. 1:**
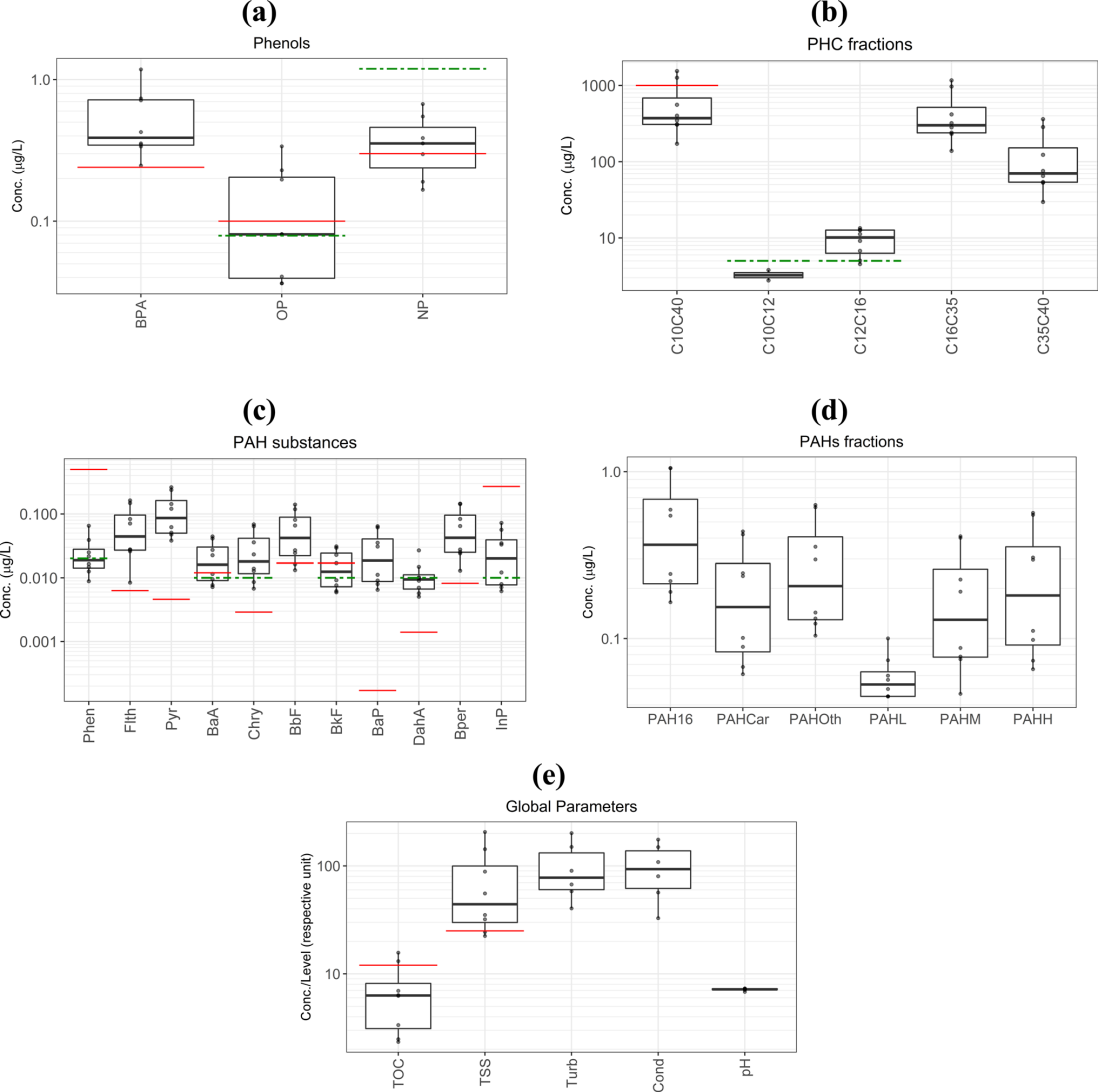
Stormwater quality parameters during all rain events (Boxplots show the 2.5, 25, 50, 75, and 97.5 percentiles of all calculated *EMCs*); red lines represent the water quality objectives (WQOs), while green dot-dashed lines illustrate the maximum censored level (if any) for the OMPs. TOC and TSS in mg/L; conductivity (Cond) in µS/cm; and turbidity (Turb) in NTU

A statistical summary (min, mean, max, SD, number of detects, and non-detects) of the best *EMCs* of various OMPs in stormwater is presented in Table 3 for all rain events. Also, the distributions of *EMCs* are depicted in Fig. 1 (a) − (e).

**Table 3:** A statistical summary of the calculated *EMC* values for all events (*N* = *n*_detected_ + *n*_censored_) at different sampling points

		SW									
Parameter	Unit	*N* (Tot.)	*n* (detected)	*n* (censored)	*EMC*_min	*EMC*_mean	*EMC*_max	*SD*	Water quality objective (WQO)*	Num. of *EMCs certainly* above WQO	Num. of *EMCs uncertainly* above WQ
Nap	µg/L	**8**	**0**	**8**			< 0.03	−	2	0	0
Acyl	µg/L	**8**	**0**	**8**			< 0.01	−	1.3	0	0
Acen	µg/L	**8**	**0**	**8**			< 0.01	−	3.7	0	0
Flu	µg/L	**8**	**0**	**8**			< 0.01	−	0.25	0	0
Phen	µg/L	**8**	**5**	**3**	< 0.015	0.026	0.065	0.017	0.5	0	0
Anth	µg/L	**8**	**0**	**8**			< 0.01	−	0.1	0	0
Flth	µg/L	**8**	**8**	**0**	0.008	0.069	0.163	0.059	0.0063	7	**1**
Pyr	µg/L	**8**	**8**	**0**	0.038	0.12	0.262	0.088	0.0046	8	0
BaA	µg/L	**8**	**6**	**2**	< 0.008	0.021	0.044	0.014	0.012	4	0
Chry	µg/L	**8**	**7**	**1**	< 0.009	0.029	0.069	0.023	0.0029	7	**1** (censored)
BbF	µg/L	**8**	**8**	**0**	0.013	0.061	0.14	0.049	0.017	6	2
BkF	µg/L	**8**	**6**	**2**	< 0.006	0.016	0.031	0.01	0.017	3	**1**
BaP	µg/L	**8**	**8**	**0**	0.006	0.028	0.064	0.024	0.00017	8	0
DahA	µg/L	**8**	**5**	**3**	< 0.006	0.011	0.027	0.006	0.0014	5	**3** (censored)
Bper	µg/L	**8**	**8**	**0**	0.013	0.066	0.145	0.054	0.0082	8	0
InP	µg/L	**8**	**7**	**1**	< 0.006	0.029	0.072	0.023	0.27	0	0
∑16 PAHs	µg/L	**8**	**8**	**0**	0.165	0.507	1.052	0.372	−	−	−
∑Car PAHs	µg/L	**8**	**8**	**0**	0.061	0.208	0.438	0.155	−	−	−
∑non-Car PAHs	µg/L	**8**	**8**	**0**	0.104	0.3	0.631	0.218	−	−	−
∑LMW PAHs	µg/L	**8**	**8**	**0**	0.045	0.059	0.1	0.019	−	−	−
∑MMW PAHs	µg/L	**8**	**8**	**0**	0.047	0.19	0.41	0.147	−	−	−
∑HMW PAHs	µg/L	**8**	**8**	**0**	0.066	0.258	0.566	0.208	−	−	−
BPA	µg/L	**8**	**8**	**0**	0.247	0.542	1.179	0.314	0.24	7	**1**
OP	µg/L	**8**	**6**	**2**	< 0.041	0.131	0.338	0.104	0.1	3	0
OP1EO	µg/L	**6**	**0**	**6**			< 0.024	−	0.9	−	−
OP2EO	µg/L	**6**	**0**	**6**			< 0.45	−	0.91	−	−
OP3EO	µg/L	**6**	**0**	**6**			< 0.033	−	0.91	−	−
NP	µg/L	**8**	**5**	**3**	< 0.166	0.374	1.19	0.181	0.3	3	**3** (2 censored)
NP1EO	µg/L	**6**	**0**	**6**			< 0.18	−	0.64	−	−
NP2EO	µg/L	**6**	**0**	**6**			< 2.54	−	0.37	−	−
NP3EO	µg/L	**6**	**0**	**6**			< 1.27	−	0.3	−	−
C_10_ − C_40_	µg/L	**8**	**8**	**0**	172.18	613.19	1545.56	506.12	1000^¥^	−	−
C_10_ − C_12_	µg/L	**8**	**2**	**6**	< 2.772	3.283	5	0.512	900^$^ (Arom.), 300^$^ (Aliph.)	−	−
C_12_ − C_16_	µg/L	**8**	**7**	**1**	< 4.551	9.375	13.392	3.445	900^$^ (Arom.), 300^$^ (Aliph.)	−	−
C_16_ − C_35_	µg/L	**8**	**8**	**0**	138.6	470.96	1167.74	380.85	900^$^ (Arom.)	−	−
C_35_ − C_40_	µg/L	**8**	**8**	**0**	29.67	131.00	361.55	123.47	−	−	−
TOC	mg/L	**8**	**8**	**0**	2.34	10.06	23.03	7.08	12^¥^	8	0
TSS	mg/L	**8**	**8**	**0**	2.37	66.51	205.70	60.60	25^£^	9	**2**
Turb	NTU	**8**	**8**	**0**	40.39	107.17	201.11	54.79	−	−	−
EC	µS/cm	**8**	**8**	**0**	32.77	138.39	299.49	82.08	−	−	−
pH	**-**	**8**	**8**	**0**	6.799	7.148	7.308	0.156	6.5–9^¥^	−	−
Temp	°C	**8**	**8**	**0**	11.62	16.52	22.35	3.38	−	−	−

*The water quality objectives refer to the lowest Predicted No-Effect Concentrations (PNEC) in the NORMAN Ecotoxicology Database and/or the Annual Average Environmental Quality Standards (AA EQS) in the European Union Water Framework Directive (2013/39/EU;WFD); both serve as established standards for freshwater quality unless another guideline is mentioned.

^¥^Gothenburg’s stormwater guideline for water recipients (Miljöförvaltningen 2013)

^$^WHO’s guideline for aromatic (Arom.) and aliphatic (Aliph.) PHCs in drinking water (WHO 2008)

^£^Protective threshold against chronic effects in freshwater set by European Council (EC 2006)

According to the results of stormwater quality analysis for eight rain events, BPA, OP, and NP were detected in the runoff from 100, 75, and 66% of all rain events (Table 3), respectively. In rain events C, F, G, and H, these three phenolic substances were detected simultaneously. Among the phenolic substances, the *EMCs* of BPA and NP were higher than what was measured for OP (Fig. 1 (a)). The *EMC* of BPA varied from 0.247 and 1.179 µg/L, with a median concentration of 0.39 µg/L. The *EMC* values of NP were in a similar range (< 0.166–1.19 µg/L, with a median concentration of 0.34 µg/L). The corresponding values for OP were < 0.041–0.338 µg/L (median: 0.08 µg/L). Unlike phenolic substances, nonylphenol and octylphenol ethoxylates (NPnEO and OPnEOs; *n* = 1,2,3) were never quantified in the road stormwater samples analyzed in this study (always ∑NPnEO < 2.54 and ∑OPnEO < 0.45 µg/L; see Table 3 for more details).

PAHs were frequently quantified in the highway stormwater samples. Some PAH substances, including Flth, Pyr, BbF, BaP, and Bper, were detected across all rain events, while Chry and InP were detected in 87%, BaA and BkF in 75%, DahA in 62%, and Phen in 50% of events (Table 3). On the other hand, Nap, Acy, Ace, Anth, and Flu (all among LMW PAHs) were never quantified in the assessed stormwater samples (Table 3). The ∑16 PAH concentrations varied between 0.165 and 1.052 μg/L, with a median *EMC* of 0.37 μg/L. Carcinogenic PAHs contributed to 41% of the total PAHs. BaP, considered as the most potent carcinogenic PAH, was detected across all events at concentrations between 0.006 and 0.064 µg/L, with a median concentration of 0.019 (*SD* = 0.024) µg/L. Of the 59% of total PAHs that were non-carcinogens, about 38% was attributed to Flth and Pyr (equivalent to ∑MMW PAHs).

C_16_-C_35_ and C_35_-C_40_ were the dominant fractions of PHCs observed in the highway stormwater (Fig. 1 (b)). The concentrations of all PHCs (C_10_-C_40_) ranged from 175 and 1539 µg/L, with a median *EMC* of 385 µg/L. The fractions C_10_-C_12_ and C_12_-C_16_ (i.e., lighter PHC molecules) were rarely found in the stormwater samples, with relatively low maximum concentrations of 5 and 13 µg/L, respectively.

Finally, the analysis of global parameters in the sampled stormwater revealed that TSS ranged from 22.4 to 206 mg/L (median: 54 mg/L), while TOC ranged from 2.3 to 23.0 mg/L (median: 7.1 mg/L). The pH value of the stormwater remained in the neutral range of 6.7–7.3, as was expected. Onsite measurements also showed that the turbidity of samples ranged from 40.4 to 201.1 NTU for all rain events.

The following section will describe the observed stormwater quality by comparing our results with the measured levels of OMP reported in other similar studies as well as water quality objectives.

#### Phenolic substances

Phenolic substances were often quantified in the highway stormwater, with BPA being the most frequently observed substance, followed by OP and NP. Although the variable LoQs for OP and NP caused some uncertainties in the results (discussed in “Uncertainty analysis of EMC calculations using a MC method”), both of these compounds were detected in more than two-thirds of the rain events. Similarly, a recent metastudy on micropollutants also demonstrated that BPA and OP are often detected in > 75% of stormwater flows, whereas NP is observed less frequently, but demonstrates a higher risk according to Mutzner et al. (2022). The median *EMC* estimated for BPA was either similar or approximately half of what has previously been measured by other researchers in runoff from major roads (see Table 4). The *EMC* values of NP and OP were often similar to, or slightly lower than, what has been observed in other road catchments, but distinctly lower than what was reported by Gasperi et al. (2022) (see Table 4). The primary sources of BPA, NP, and OP in roadways are traffic-related materials (brake fluids, car bodies, and tires) and infrastructural chemicals (asphalt modifiers and road paints (Lamprea et al. 2018; Markiewicz et al. 2017). Accordingly, the observed concentration differences between our study and two prior studies can be explained by a higher traffic load (1.7–3 times), which would cause a higher pollutant load than what was experienced in the two other studies; the noticeable differences in TSS levels (see Table 4) also support this hypothesis. Secondly, additional sources of NPs (e.g., varnishes, lacquers, water paints, floor coatings, coated metal surfaces, polymeric compounds) might exist in one of the catchments with mixed urban runoff studied by Gasperi et al. (2022) relative to a mere roadway catchment.

**Table 4:** Comparison of the concentration ranges of stormwater OMPs (min-median-max) observed in this study and other field studies

	Reference	Catchment type	Traffic load (veh/d)	∑PAHs (µg/L)	PHCs (mg/L)	BPA (µg/L)	NP (µg/L)	OP (µg/L)	∑NPnEOs (µg/L)	∑OPnEOs (µg/L)	TSS (mg/L)	TOC (mg/L)
Sweden	**This study**	E4 highway/ major road	13,000	(0.16–0.37–1.1)	(0.17–0.4–1.5)	(0.25–0.39–1.2)	(< 0.17–0.34–1.2)	(< 0.04–0.08–0.34)	(< 4)	(< 0.51)	(2.4–54–206)	(2.3–7.1–23)
	Markiewicz et al. 2017**)**	Motorway	NA^$^	Max available: 9–190 (≈5.8–29 kg/ha·yr)	–	–	–	–	–	–	–	–
	Nielsen et al. (2015)	Motorway	40,000	(3.2–139)	–	–	–	–	–	–	(130–150–170)	–
	Kalmykova et al. (2013)	Motorway + industrial + landfill	85,000 (motorway)	(0.02–0.32–12.1) (LMW > 80%)	–	(< 0.01–0.5–107)	(< 0.1–0.3–7.3)	(< 0.01–0.23–1.3)	(< 0.1– < 0.1–3.6)	(< 0.01–0.03–5.3)	(2–820)	10–11
	Kalmykova et al. (2013)	Only motorway	85,000	Mean: 0.3 In 2005: (0.97–2.8)	–	Mean: 0.827	Mean: 1.1	Mean: 0.82	< 0.01	Mean: 9.64	9.5–350	14–20
	Björklund et al. (2009)	E6 highway	85,700	–	–	–	(< 0.1–0.26–1.2)	–	(0.2–1.3–2.2)	–	(97–150–310)	(7–11–16) (DOC > 85%)
	**(**Pettersson et al. (2005)	Motorway	NA	2.6 and 6.9	–	–	–	–	–	–	–	–
France	Gasperi et al. (2022)	Urban road (dense area)	40,000	~ (0.8–3.2–9.5)	~ (0.4–1.7–2.9)	~ (0.7–2.4–7)	~ (0.6–2–6)	~ (0.1–0.8–1.8)	~ (0.13–0.82–4.04)	~ (0.07–0.23–0.95)	(26–223–1620)	DOC: (4–15–95)
	Gasperi et al. (2022)	Suburban road	22,000	~ (1.2–5.5–13)	~ (0.11–1.4–4.7)	~ (0.2–0.4–1.1)	~ (0.4–1.9–5.3)	~ (0.18–0.5–0.7)			(54–210–933)	DOC: (2–6–15)
	Flanagan et al. (2019a, b** (2018)**	Highway in industrial zone	22,000	(1.3–4.9–11.5)	(0.3–1.1–4)	(0.2–1–4.1)	(0.9–1.6–5.8)	(0.2–0.4–1.5)	(0.21–0.59–3.57)	(0.03–0.06–0.35)	(70–291–933)	(14–49–111) (DOC: 13%)
	Leroy et al. (2016)	Roads in commercial area	2500	(0–3.2–6.5) (20% Phen)	(< 0.05–0.6–1.3)	–	–	–	–	–	(75–290–774)	(13–37.6–109)*
	Gasperi et al. (2014)	Major roads in urban areas	10,000–60,000	Mean: 0.9–1.4	–	(0.2–0.55–0.8)	(0.2–0.4–0.5)	(0.04–0.06–0.07)	(0.12–0.51–0.57)	(0.013–0.036)	Mean: 100–150	(10–32–56) (DOC < 20%)
	Bressy et al. (2012)	Mixed urban area	NA (< 2000)	(0.55–1.1–2.2)	–	–	(0.16–0.5–0.9)	(0.01–0.04–0.07)	–	–	(15–26–64)	(9–14–35) (DOC: 50%)
	Zgheib et al. (2012)	Big, dense urban area	NA (> 15,000)	(0.67–1.3–6.5)	–	–	(0.3–0.75–9.2)	(< 0.05–0.1–0.26)	–	–	(11–106–430)	(2–27–104)*
Germany	**J. **Zhang et al. (2017)	Roadway	13,600–15,000	Mean: 0.27–0.66 (≈15.9 µg/m^2^)	–	–	–	–	–	–	(41–85) (≈1–5 g/m^2^)	–
	Wicke et al. (2021)	Major road	> 7500	(0.05–4.1–11)	–	< 0.2	–	< 0.4	–	–	(1–368–1330)	(25–79–98)*
	Stachel et al. (2010)	Highways	63,000–101,000	(1.1–2.3–5.1)	C10C13: < 0.50	(0.24–1.4–2.5)	(0.2–0.8–3.6)	(0.15–0.3–1.9)	–	–	Mean: 12	–
USA	Masoner et al. (2019)	Mixed urban area	NA	(0.01–1.0–10)	–	(0.05–0.3–2.0)	–	–	–	–	–	–
	David et al. (2015)	Parking lot + public area	–	(0.67–2.3–4.6)	–	–	–	–	–	–	(2.9–21–43)	–
	Diblasi et al. (2009)	Roadway + parking lot	NA (< 2000)	(0.67–2.3–4.6)	–	–	–	–	–	–	(16–33–68)	–
*Estimated from COD concentrations ^$^Not available												

The observed *EMC* values of NP and OP were also lower than what was previously reported for Swedish and German highways. The larger daily traffic load (five times larger than the present study) in the prior Swedish study (Kalmykova et al. 2013) was probably the primary reason for the discrepancy. In the case of the German study, which investigated a road with a similar traffic load (Stachel et al. 2010), the discrepancy may be explained by the decreased use of phenolic compounds after these substances were identified as priority pollutants by the EU WFD (2013). In contrast, the OP concentrations measured in the present study were higher than what has previously been reported for diverse urban catchments (Bressy et al. 2012; Gasperi et al. 2014). This may be explained by the fact that tire wear is the primary source of OP (Flanagan et al. 2019b; Hannouche et al. 2017). The high observed OP levels may also raise the question of additional sources at the study site, such as collection pipes made of high density polyethylene (HDPE), which may release phenolic substances, including OP, into the stormwater. Further investigation is needed to clarify this discrepancy in OP concentrations. Concerning the surface WQOs based on European guidelines, the AA EQS and PNEC defined for BPA, NP, and OP (0.24, 0.3, and 0.1 µg/L, respectively) were exceeded eight, four, and three times across the eight studied rain events.

In contrast to previous studies (including those conducted in Sweden; Table 4), NPnEOs and OPnEOs (*n* = 1,2,3) were not detected in the runoff. A potential explanation could be matrix interference during chemical analysis, which adversely affected the LoQs; subsequently, we could not compare the actual concentrations with those reported by other researchers (Table 4). The reported LoQs of NPnOEs (up to ~ 20 times greater than the initial report limit in the analysis package) were affected more than the LoQs of OPnOEs (up to ~ 3 times larger). Another possible explanation is that these compounds are far more rare in roadways than in mixed urban catchments. This hypothesis is supported by the levels reported in Table 4 for these two distinct catchment types. In roads, NP- and OP-ethoxylates are mainly emitted by fuel and lubricant oils, car bodies, modified bitumen membranes, and concrete sidewalks, while in urban areas these compounds could also be released from PVC materials and polymeric resins used as lacquers, varnishes, and paints for roofing, building facade, and corrosion protection (Lamprea et al. 2018). From the perspective of freshwater quality objectives, all of the maximum levels reported for OPnEOs and NPnEOs were sufficiently below the lowest PNEC, except for NP2EO and NP3EO, with the NP2EO concentrations more of a concern (Table 3).

#### PAHs

All MMW polycyclic aromatic hydrocarbons (PAHs) and most HMW PAHs were detected in at least 50% of the rain events, while most LMW PAHs were not detected. Similarly, Mutzner et al. (2022) concluded that the PAHs observed in the present study are not only the most common and risky substances among all PAHs, but also present at noticeable levels among a wider range of micropollutants in various stormwater flows. Our results agreed with other studies in that LMW PAHs are less likely to be detected in stormwater (Järlskog et al. 2021). The LMW PAHs that were not detected may have degraded after being released into the environment due to their natural characteristics. These compounds have much greater volatility at warmer temperatures (10^−3^ < vapor pressure < 10 Pa at 25 °C) and relatively smaller sediment adsorption coefficients (2.5 < Log *K*_OC_ < 3.7) than other PAH fractions (Hawthorne et al. 2006; Khodadoust et al. 2005) (see Table S2 for more info). Therefore, they are less likely to exist in runoff water after being released. The ∑16 PAH concentrations fell within the range expected for major roads (0.03 − 6 μg/L) that was previously presented by Lundy et al. (2012). Järlskog et al. (2021) also found that LMW PAHs in stormwater are temperature-dependent, which means that they readily evaporate into the atmosphere during warmer sessions (i.e., the sampling period in this study).

However, the range of ∑16 PAHs was less than what has been reported in other studies of road catchments, either with high or lower traffic loads (see Table 4). Since most PAHs are predominantly found in the solid phase or absorbed by sediments (Markiewicz et al. 2017; Nielsen et al. 2015), it could be that the relatively low TSS levels observed in this study are an explanation for the result. This may be partly explained by the partial deposition of stormwater sediments along the transport pipe (low slope of 0.5%) from the catchment to the facility downstream. Among the detected PAHs, only Nap and InP were measured at levels that consistently fell below the lowest PNEC (AA EQS). The other PAHs were always detected at concentrations that exceeded the guideline quality objectives for freshwaters; exceptions were BaA, BbF, and BkF, which exceeded quality objectives in 50, 20, and 40% of rain events, respectively.

In the studied catchment, the PAHs most likely originated from vehicular traffic (brake lines, tire wear, exhaust gases/particles, and engine oil (Burant et al. 2018; Kose et al. 2008; Markiewicz et al. 2017)) and the abrasion of pavement material by tires and runoff water on the old asphalt road and sidewalks (Crane 2014; Müller et al. 2020). However, regarding the pavement surface wear, it should be noted that the usage of tar coal asphalt (containing PAHs) has been legally forbidden in Sweden since 1973. So, the worn asphalt concrete material of the E4 highway bride (built in 2014) should not be a source of PAHs itself. Besides, assuming the maximum *EMC* for Phen (the only LMW PAH detected) and the minimum and maximum concentration values for ∑HMW PAHs, the results revealed a PAH diagnostic ratio Σ(LMW)/Σ(HMW) between 0.04 and 0.1, which is much lower than one. This means that PAHs in the road stormwater are more likely to originate from pyrogenic (vehicular emissions from exhaust after petroleum or liquid fossil fuel combustion) rather than petrogenic (e.g., brakes, vehicle tire debris, and spilled engine oil) sources. This finding agrees with previous literature on road runoff quality (Li & Kamens 1993; Markiewicz et al. 2017; Szopińska et al. 2019; J. Zhang et al. 2017).

Moreover, rain events B, C, E, and H demonstrated the highest load of PAH pollution, both in terms of diversity and concentration of PAHs. No particular rain characteristics (given in Table 1) could explain the PAH concentrations observed during these rain events. Rain events B and C with the highest observed PAH levels occurred in the time periods before and after winter conditions at which vehicles having winter tires (with more grip) may hypothetically cause greater tire wear particles on the roads with no ice/snow cover. However, further evidence and research are needed to demonstrate this.

#### PHCs

The *EMC* range for total petroleum hydrocarbons PHCs (C_10_-C_40_) was similar as what was reported by Gasperi et al. (2022) and Leroy et al. (2016), yet half to one order of magnitude lower than what was observed in runoff from roads with low and high traffic loads, respectively (Järlskog et al. 2021; Kayhanian et al. 2007; K. Zhang et al. 2014) (see Table 4). Heavier PHC fractions, namely, C_16_-C_35_ and C_35_-C_40_, dominated the measured PHCs. The low-weight PHC molecules were most likely found at lower levels relative to heavier PHC molecules because aromatic/aliphatic C_10_-C_16_ have higher volatility and much lower Koc than heavier counterparts (> C_16_). On the other hand, aromatic/aliphatic compounds above C20 are neither volatile nor soluble in the aquatic phase and are thus likely to be absorbed by suspended solids (Reed & Stemer 2002). However, higher-weight PHC molecules are usually considered to be less toxic for organisms, which means that > C_20_ compounds in the runoff represent a lower health risk. The observed *EMCs* for total PHCs often fell below 1000 μg/L, which is the city of Gothenburg’s local guideline recommendation for releasing waters polluted with aliphatic and aromatic C_10_-C_40_ (Järlskog et al. 2021; Miljöförvaltningen 2013); this threshold was exceeded in 25% of rain events. However, the levels are still a matter of concern given WHO drinking water standards (WHO 2008) (Table 3).

The two most frequently PHC fractions (C_16_-C_35_ and C_35_-C_40_) were detected across all rain events, but the highest concentrations occurred during events B, C, E, and H; these rain events also demonstrated the highest levels of PAHs. Similarly, the highest PHC concentrations were recorded at rain events B and C. Again, this might be related to the presence of more winter tire (with/without stud) and asphalt wear particles at those times of the year when no ice/snow has covered the road surfaces. Though, this hypothesis requires further investigations.

#### Global parameters

Although the *EMCs* of TSS were near the low end of globally-observed mean values on major roads (110–5700 mg/L according to Lundy et al. (2012)), they still surpassed the European quality objective of 25 mg/L (the protective threshold against chronic effects in freshwater (EC, 2006)) across 80% of the rain events. When compared to the scientific literature (Table 4), the TSS and TOC values were comparable with what has been observed in runoff from Swedish/European/North-American motorways, but noticeably lower than what has been reported for some roads with higher traffic loads (Flanagan et al. 2018; Gasperi et al. 2014; Kayhanian et al. 2012). The notable difference may be explained by site-specific conditions (e.g., an extended acceleration zone, a higher proportion of heavy trucks, and lower precipitation over the study period), which would cause high wear particle production and more concentrated runoff (Flanagan et al. 2018). Meanwhile, we must also assume that the 50-m-long transportation pipe in our site may have attenuated stormwater TSS load to a certain degree before the runoff reached the SW sampling point; this would be especially relevant during less intense rainfall.

### Uncertainty analysis of EMC calculations using a MC method

The recalculation of *EMC* values through MC simulation involved uncertainty analysis to get a reliable picture of OMP levels. As previously mentioned, each calculated *EMC* is associated with uncertainties due to analytical and sampling constraints; these uncertainties may influence data accuracy and interpretation. Analytical errors are a significant source of uncertainty for research into micropollutants because these compounds usually exist at low concentrations (Flanagan et al. 2018). Before the start of a rain event, the stormwater present in the downstream GPT chamber (which was used to calculate flow volume at the study site) was another source of uncertainty, as it affected the first sampled volume (i.e., the first 23.3 m^*3*^ or *v*_1_). However, the relatively large total number of subsamples or sub-subsamples (smaller portions of a single subsample taken by a number of serial pulses) collected during the whole event decreased the influence of *v*_1_ on *EMC* errors. In the case of rain events A, B, C, and D (which comprised fewer total number of subsamples than the other four rain events), at least five pulses were received from the discharging valve during the first sample collection (5 sub-subsamples for *c*_1_; total no. pulses > 10). The total number of received pulses during rain events E, F, G, and H exceeded 12. Therefore, *EMC* errors due to *v*_1_ were negligible, compared with those due to analytical uncertainty, which was the primary source of uncertainty in the present study.

**Fig. 2:**
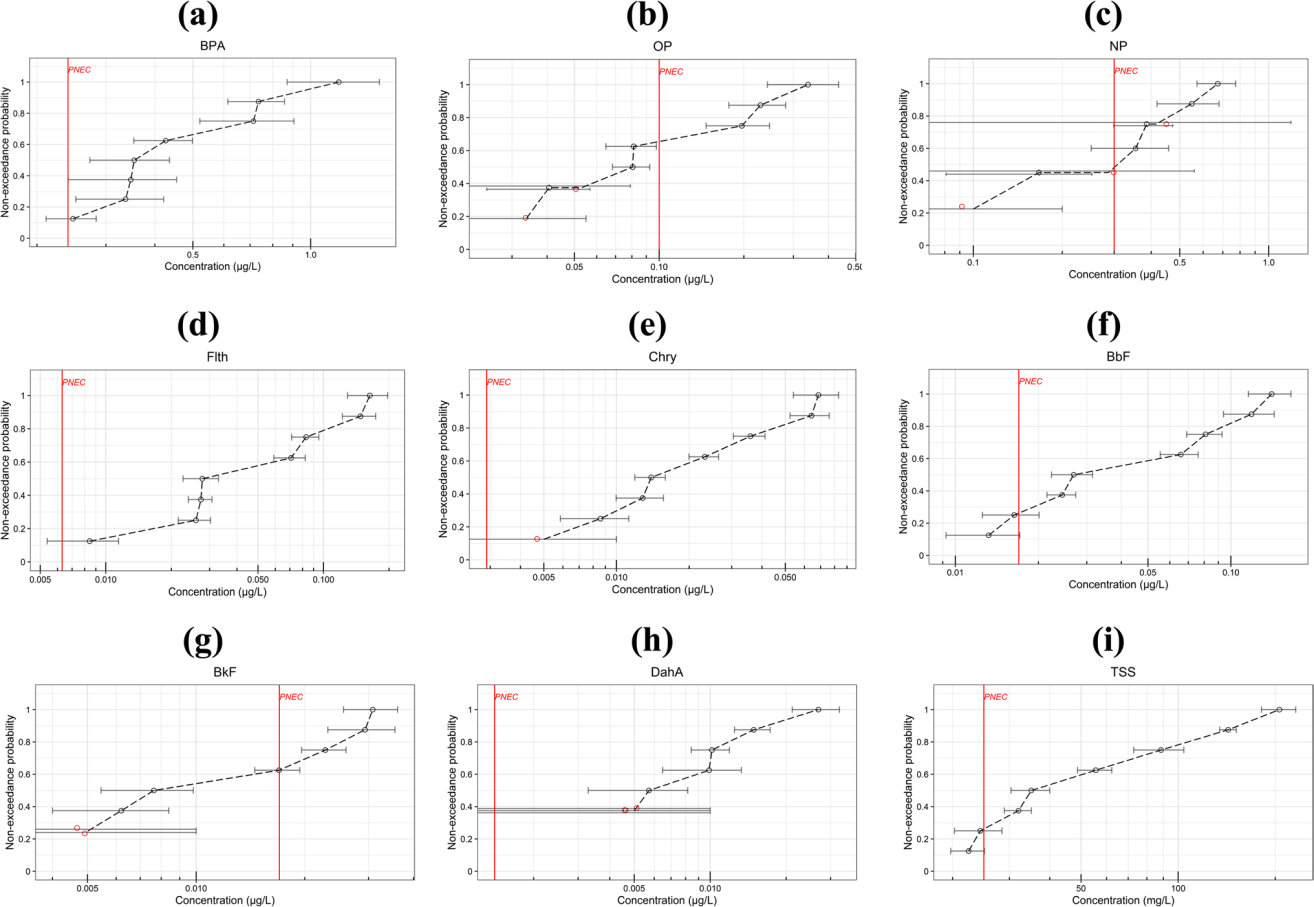
Non-exceedance probability (NEP) plots of the estimated *EMCs* for each OMP (black points are detects and red points censored data (non-detects); error bars show *EMC* errors (uncertainties); red lines represent the lowest PNEC levels for freshwater, based on water quality objectives (WQOs))

**Fig. 3:**
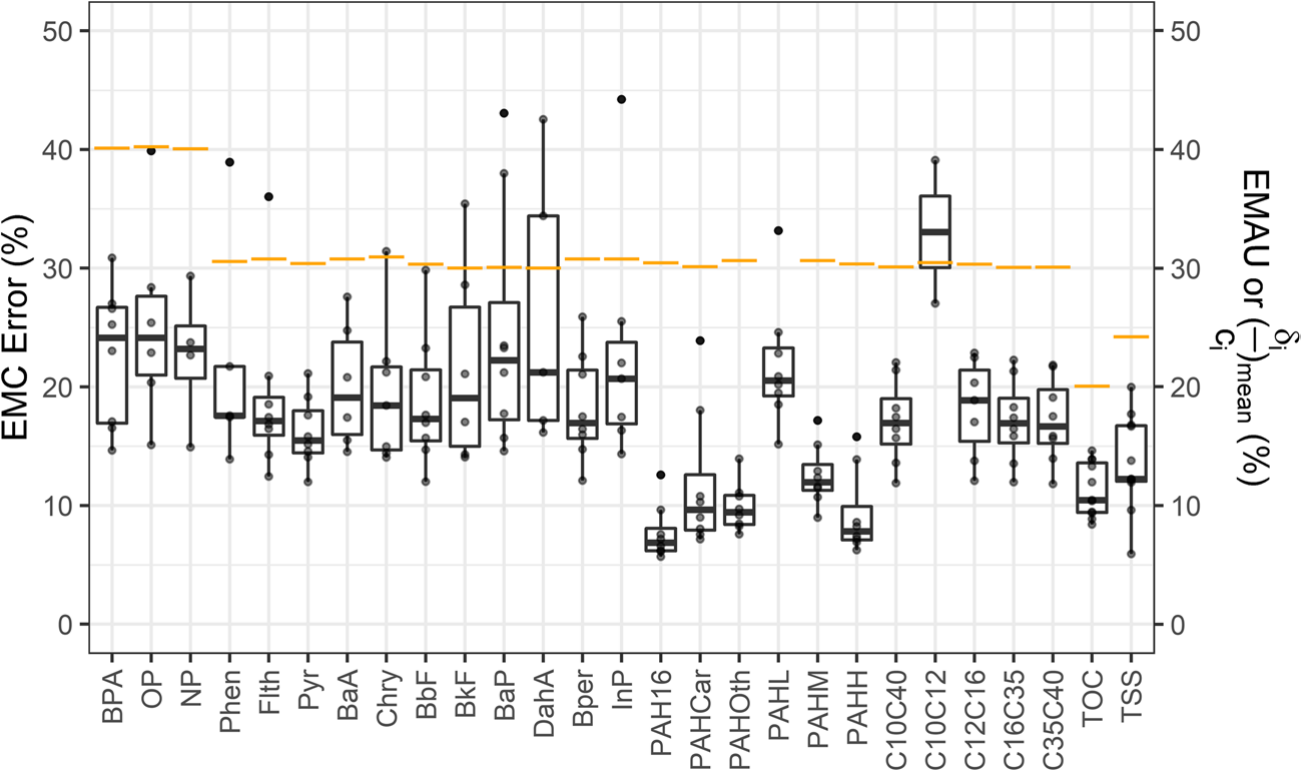
Statistical analysis of *EMC* estimation errors (boxplots: min, *Q*_25%_, *Q*_50%_, *Q*_75%_, and max of relative *EMC* errors over *N* = 8 events) (orange bars: mean of relative analytical uncertainty observed for all events (*EMAU* or (*δ*_*i*_/*c*_*i*_)_mean_))

To assess *EMC* errors and their impact on data interpretation, non-exceedance probability (NEP) plots were generated for the *EMC* values of each OMP. A NEP plot does not only indicate the distribution of best-estimated *EMCs* across all rain events, but also visualizes the *EMC* ranges (*EMC*–*Δ*_*l*_, *EMC* + *Δ*_*u*_) resulting from uncertainty propagation in the MC method. The NEP plots for selected OMPs are presented in Fig. 2 (a) to (i) (the rest shown in Fig. S2 (a) to (t)). The NEP plots also revealed which *EMCs* might have reached the corresponding PNEC levels (WQOs) due to uncertainties (the number of rain events during which the threshold was exceeded due to uncertainty is shown for each OMP in Table 3). This was the case for BPA, NP, Flth, BbF, BkF, and TSS at one or two events (Fig. 3). There were also a few events during which the censored concentrations of NP, Chry, and DahA (censoring as the only uncertainty factor) might have exceeded PNECs (see Fig. 3).

To further assess uncertainty levels, a statistical summary of relative *EMC* errors among all of the rain events is shown in Fig. 3 for OMPs, TSS, and TOC. An evaluation of the lower and upper uncertainties of the calculated *EMCs* revealed that all *EMCs* possessed approximately symmetric distribution (< 1% difference between left and right *EMC* errors), which predominantly followed the symmetric behavior of the *normal* distribution of analytical uncertainties. Therefore, the reported errors in Fig. 3 represent the *EMC* errors for both sides (|± *Δ*| (%)).

The MC method-facilitated analysis of errors generally showed that the *EMC* errors for OMPs can vary between 5 and 45% (median errors between 10 and 25%, with the exception of 35% for C_10_-C_12_). Comparing different groups of OMPs (Fig. 3) also revealed that phenolic substances had the highest *EMC* error range (23 − 25%), which means that the reported levels of these compounds in the studied stormwater are associated with less reliability. The median *EMC* errors for PAH substances, and PHCs were in between the range of 17 − 23% (except for C_10_-C_12_ due to censored data). We also observed that the *EMCs* of TOC and TSS were associated with the smallest errors (10% and 12%, respectively). The observed differences in errors can be linked to differences in the corresponding analytical uncertainties (*δ*_*i*_); in this way, higher *δ*_*i*_ values resulted in larger *EMC* errors. To compare the levels of such uncertainties among the investigated OMPs, all-events-mean relative analytical uncertainty (*EMAU* or (*δ*_*i*_/*c*_*i*_)_*mean*_, *i* = subsample number) was calculated and defined as an analytical uncertainty index for each OMP (orange bars in Fig. 3). So, as implied in Fig. 3, *EMC* error boxplots can change proportionally to *EMAU* variations. Furthermore, the median *EMC* errors were considerably less than the calculated *EMAU* values; this indicates how the original measurement uncertainties (*δ*_*i*_) affected the calculations, i.e., in the *EMC*_best_ estimation using MC, the *EMC* error (or the standard error of the mean, which is a function of subsample standard deviations (~ *δ*_*i*_*/*2)) declines based on the square root of the number of subsamples (Feiguin 2009). It should also be noted that the errors of the PAH-H and PAH-M fractions (median: 12 − 15%, except PAHL) were lower than those for PAH substances (median: 17 − 23%), although both showed similar *EMAU*. This was expected, as the *EMCs* for PAH fractions were estimated from the substance concentrations after applying the MC method twice (see “EMC estimation and uncertainty propagation using a Monte-Carlo method”).

Thus, our error analysis revealed a considerable degree of uncertainty in the organic pollutant data; this is important to keep in mind when interpreting the data of other studies. We also found that, as expected, increasing the amount of censored data among subsamples increased the *EMC* error (see e.g., BkF, DahA, PAHL, C_10_-C_12_, OP, and NP in Fig. 3), which makes it hard to draw an appropriate conclusion regarding the *EMC* levels in those cases. Besides, it should be noted that although the applied MC simulation approach attenuated uncertainty in the actual measurements by about 10 to 15% (towards more optimistic results), this approach can be used as a simple and reliable method for estimating the *EMCs* of OMPs.

### Correlated water quality parameters

Using statistical correlation analyses, we attempted to understand the mathematical relationships between the studied parameters with the objective of possibly identifying correlated conventional water quality parameters and rain characteristics that alongside monitoring campaigns can help predict various highway runoff OMPs in this specific site. So, continuously measuring those conventional parameters could potentially complement data from monitoring programs in which long-term, high-resolution time series are of interest. The results of correlation analyses for all parameters are summarized in Table 5. Only the correlation coefficients (*rho* or *tau*) that demonstrated statistical significance (*p*-value ≤ 0.05) are included in this table. The entire correlation matrix, including confidence levels, can be found in Table S4.

**Table 5:** Summary of significantly correlated parameters

OMP categories	Correlated parameters	Correlation coefficient	Statistical test
Phenolic substances	BPA and TOC	+ 0.88	Spearman
	OP and some PAHs^*^	+ (0.61–0.68)	Kendall’s tau
	OP and 16PAHs	+ 0.86	Kendall’s tau
	OP and Tot. PHCs	+ 0.68	Kendall’s tau
	OP and C_16_-C_35_	+ 0.68	Kendall’s tau
	NP and I_P_	+ 0.5	Kendall’s tau
PAHs	Pairs of PAHs	+ (0.74–0.98)	Spearman
		+ (0.57–0.74)	Kendall’s tau
	PAHL and PAHM	+ 0.86	Spearman
	PAHL and PAHH	+ 0.81	Spearman
	PAHM and PAHH	+ 0.98	Spearman
	PAHCar and PAHNon-car	+ 0.95	Spearman
	Some PAHs^₹^ and turbidity	+ (0.83 − 0.94)	Spearman
	Some PAHs^¥^ and turbidity	+ (0.73 − 0.8)	Kendall’s tau
	Some PAHs^€^ and TSS	+ (0.76–0.88)	Spearman
	Phen and TSS	+ 0.57	Kendall’s tau
	PAHCar and turbidity	+ 0.89	Spearman
	16PAHs and turbidity	+ 0.94	Spearman
	16PAHs and TSS	+ 0.74	Spearman
PHCs	Tot. PHCs and C_16_-C_35_	+ 1	Spearman
	Tot. PHCs and C_35_-C_40_	+ 0.91	Spearman
	C_16_-C_35_ and C_35_-C_40_	+ 0.91	Spearman
	Tot. PHCs^£^ and some PAHs^$^	+ (0.68–0.82)	Kendall’s tau
	Tot. PHCs^£^ and some PAHs ^#^	+ (0.79–0.98)	Spearman
	Tot. PHCs and turbidity	+ 0.94	Spearman
	C_16_-C_35_ and turbidity	+ 0.94	Spearman
	C_35_-C_40_ and turbidity	+ 0.84	Spearman
	C_35_-C_40_ and TSS	+ 0.74	Spearman
Global	TSS and turbidity	+ 0.9	Spearman
	TOC and EC	+ 0.78	Spearman
	TOC and ADP	+ 0.65	Spearman

^*^Pyr, BaA, Chry, BbF, BaP, and InP

^₹^Flth, Pyr, BbF, BaP, Bper, and Inp

^¥^Phen, BaA, and BkF

^€^Flth, Pyr, and BbF

^$^Phen, BaA, Chry, BkF, DahA, and InP

^#^Flth, Pyr, BbF, BaP, and Bper

^£^Predominantly C_16_-C_35_ and C_35_-C_40_ fractions

The results did not reveal any correlations between TOC and TSS concentrations, which suggest that organic carbon in road runoff is mainly dissolved. Other DOC and TOC measurements at the same site before our sampling period (Lange et al. 2022) support this hypothesis. This result also agrees with what was reported in previous studies from Swedish and North-American highways (Björklund et al. 2009; Kayhanian et al. 2012), but disagrees with findings reported for major roads in France (Bressy et al. 2012; Flanagan et al. 2018; Gasperi et al. 2014). An abundance of suspended particles, which readily absorb organic carbon, might be responsible for the contradictory partitioning in these studies (Flanagan et al. 2018). Furthermore, TOC was strongly correlated with conductivity, which may support the assumption that organic carbon mainly exists in the dissolved phase. TOC was also associated with rain depth and *I*_peak_.

Among phenolic pollutants, BPA was highly associated with TOC. Considering the previous hypothesis about TOC, this association suggests that BPA is more likely to exist in dissolved or colloidal forms in stormwater, i.e., less likely to be absorbed by suspended particles, which agrees with what was stated by Flanagan et al. (2019a) and Gasperi et al. (2022). However, Shehab et al. (2020) and Markiewicz et al. (2017) proposed contradictory dynamics. OP was strongly correlated with most PAHs, C_16_-C_35_, and Total PHCs, but not with turbidity or TSS. This may suggest that OP is released from similar sources as PAHs and heavier PHCs (e.g., tire particles), but then undergoes a different partitioning pathway (leaching in dissolved form) and/or does not attach to suspended solids, especially larger particles, as some other studies suggest (Gasperi et al. 2014; Kalmykova et al. 2013; Shehab et al. 2020). On the other hand, NP did not show any association with other parameters, which can be, in our study, related to the number of non-detected values (three out of eight events) with different levels of censoring. The lack of an association between OP and NP also supports the idea that the sources of these two compounds differ.

Within PAHs, the *EMC* values of all regularly detected substances and fractions (excluding Nap, Acy, Ace, Anth, and Flu, which were rarely detected) were highly associated with each other and turbidity and somewhat associated with TSS. As discussed before, this finding may support that many PAH fractions have similar sources and transport pathways in stormwater (Järlskog et al. 2021). Similarly, regarding PHC fractions, heavier fractions (including C_16_-C_35_ and C_35_-C_40_, total PHCs (C_10_-C_40_), TSS, and turbidity) were strongly correlated with each other. These fractions also demonstrated strong associations with PAH substances/fractions, which may suggest similar sources and/or environmental fates in stormwater. In contrast, no meaningful correlations were observed between the *EMCs* of lighter PHCs (C_10_-C_12_ and C_12_-C_16_) and the investigated parameters or other PHC fractions. This could be explained by either a high proportion of censored data (less precise statistical relationship) or lower concentrations for lighter fractions relative to heavier C fractions, which may adversely influence the peak response during analysis. The investigated PAHs and PHCs showed a higher degree of correlation with turbidity than with TSS, which means that turbidity could be a better indicator for particulate/particle-bound OMP pollution loads in stormwater than TSS. This finding may explain that the particulate PAHs and PHCs tend to stay suspended longer (finer/lighter/more stabilized particles), whereas TSS contains dense, inorganic particles which rapidly sediment from stormwater. Another reason for this result may be that PAHs and PHCs have high affiliation for finer suspended soil particles due to higher surface charge and/or higher surface area.

Although other studies have found that rain characteristics can influence the event mean concentrations of common pollutants such as metals (Kayhanian et al. 2007), our results did not show any significant correlations between rain characteristics (depth, ADP, *I*_mean_, and *I*_peak_) and OMP levels. While it was expected that turbidity and TSS, and possibly OMP loads, are dependent upon event runoff volume or intensity (Murphy et al. 2015), we did not observe any significant correlations between TSS and rain depth, *I*_mean_, and *I*_peak_; a plausible explanation for the lack of significant correlations could be the limited number of rain events included in this study (Lange et al. 2021). Thus, the impact of rain characteristics on the *EMC* values of organic micropollutants remains unclear and requires further investigation*.*

**Fig. 4:**
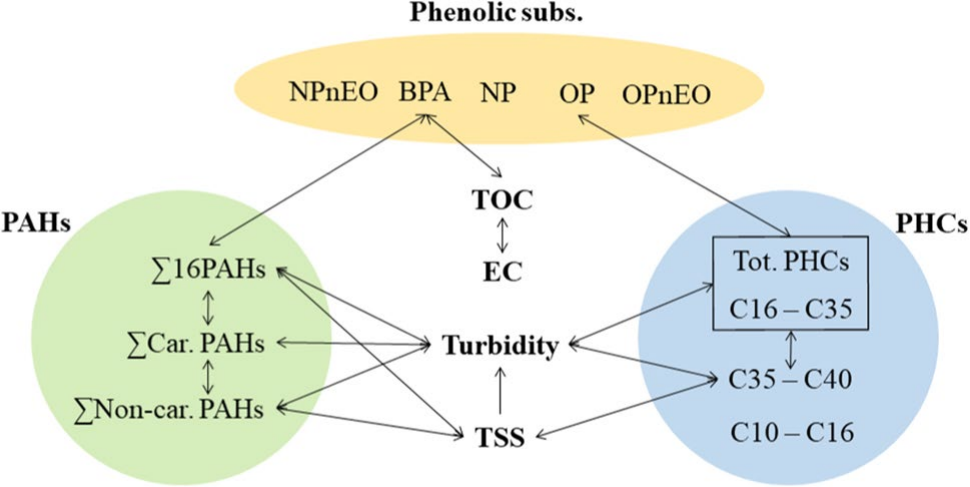
Highly correlated parameters with OMPs in the studied catchment

The relationships among all studied parameters have been depicted in Fig. 4. What these correlations suggest is that turbidity has the potential to be used as a conventional indicator parameter for estimating the contamination levels of PAHs, total PHCs, and heavier fractions of PHCs at this specific site. However, further studies are needed to establish a surrogate parameter based on these correlations. In the same way, TOC and EC could also serve as complementary quality parameters for indicating BPA levels at this site. Within the PAH category, Pyr, and BaP can also be decent indicators for non-carcinogenic and carcinogenic PAHs, respectively. Moreover, our results did not identify any correlated conventional parameters to lighter PHCs, OP, NP, and OP- and NP-ethoxylates (due to their low concentrations around LoQs).

## Conclusion

This field monitoring study demonstrated that organic micropollutants are a concern in highway stormwater runoff. The reported *EMC* values revealed that the runoff contained considerable amounts of phenolic substances, including BPA, OP, and NP (but not alkylphenol ethoxylates; OPnEO and NPnEO), with concentrations above or around corresponding EQSs for freshwater. For example, BPA was found in all of the samples within the same concentration range that was reported by other researchers. At the same time, OP and NP were detected in > 65% of samples, but often at levels lower than what have been reported in other studies around Europe. The analysis and interpretation of data concerning OP and NP were slightly affected by different censoring levels and high measurement uncertainties caused by matrix interference during laboratory analysis. The data analysis revealed that BPA was correlated with TOC (predominantly dissolved and associated with EC), while OP was found to be associated with PAH levels and the concentration of total PHCs. This finding suggests that phenolic substances may have different sources or fractionation pathways and, thus, different environmental fates and transport in road runoff.

Regarding the more common OMPs, PAHs, and PHCs were also identified in the runoff (mainly in the form of heavier weight fractions), although the observed concentrations often fell below the ranges reported in other studies performed under similar conditions. Nevertheless, the mean concentrations of MMW- and HMW-PAHs still significantly exceeded the corresponding freshwater quality objectives across most rain events. Five PAH substances (BaP, BaA, Chry, BbF, and DahA), classified as extremely or possibly carcinogenic, were observed among the risky PAHs (*EMC* > *EQS*). A diagnostic ratio analysis showed that PAHs at the studied catchment probably originate from pyrogenic sources (vehicular emissions after combustion). Statistical correlation analyses supported the fact that PAHs and PHCs (heavier fractions), which are characterized by low solubility and high stability in different phases, are associated with the levels of suspended solids (especially finer particles) in stormwater runoff.

Further statistical analyses suggested that three conventional water quality parameters, including turbidity, TOC, and EC, are strongly associated with OMPs: turbidity with PAHs, PHC, and TSS and TOC and EC with BPA. This indicates that these parameters have the potential to be used as surrogates, though such a relationship requires further work to establish and would be also site-specific.

The presented research, which provides an approach for calculating reliable *EMC* values and analyzing data to face future monitoring challenges, yielded several additional lessons for researchers:

- In the case that a certain OMP has a high proportion of censored data, a greater number of events shall be monitored, especially when the EQS is below the LoQ.
- Highly accurate chemical analysis is needed to detect analytically-sensitive OMPs, e.g., phenolic substances with variable LoQs.
- Understanding the fractionation of OMPs (particulate, colloidal, and dissolved) is beneficial, especially when choosing proper quality treatment practices.
- More in-depth research on the current sources of the OMPs in the study catchment would benefit future source management.
- Although collecting a composite sample for an entire rain event would be a better approach in such studies, MC simulation can be used to reliably estimate *EMC* values and the associated uncertainty from subsamples’ concentration and volume datasets.

## Supplementary Information

Below is the link to the electronic supplementary material.

Supplementary file1 (DOC 3438 KB)

## Data Availability

All the data generated/analyzed in this study are included in the manuscript, supplementary information. The processed dataset and the code used to generate the dataset is available at https://doi.org/10.5878/nny1-2045 .
