# Supplementary material for: Occurrence and concentrations of organic micropollutants (OMPs) in highway stormwater: a comparative field study in Sweden
Source: Environ Sci Pollut Res Int. 2023 May 31;30(31):77299–317. doi: 10.1007/s11356-023-27623-9 (PMC10299930; doi:10.1007/s11356-023-27623-9)
Supplement: Supplementary file 1 — Supplementary file1 (DOC 3438 KB) [file 11356_2023_27623_MOESM1_ESM.doc]

**Supplementary information**

# Occurrence and concentrations of organic micropollutants (OMPs) in highway stormwater: A comparative field study in Sweden

Ali Beryani^(1)(*)^, Kelsey Flanagan^(1)^, Maria Viklander^(1)^, Godecke-Tobias Blecken^(1)^

^(1)^ Department of Civil, Environmental, and Natural Resources Engineering, Luleå University of Technology, 97187 Luleå, Sweden

^(*)^ Corresponding Author: [ali.beryani@ltu.se](mailto:ali.beryani@ltu.se)


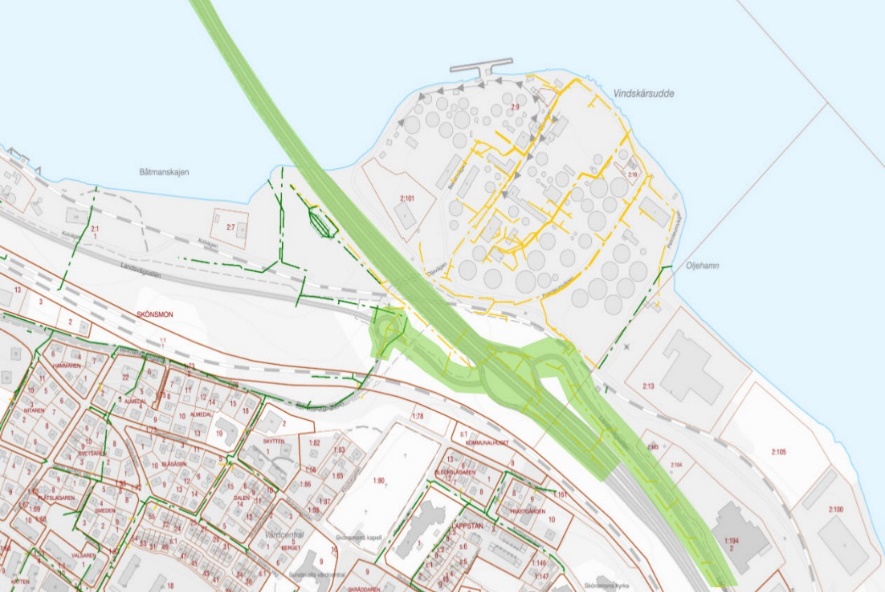


Rain gauge

Sampling point

Pipeline

100 m

Figure S1. Site plan of the catchment area (denoted in green) in Sundsvall, Sweden

Table S1. Standard analytical methods used for determining the concentrations of organic substances and other parameters

| **Parameter** | **Analytical method** |  |
| --- | --- | --- |
| **PAHs** | Determination of semi-volatile organic compounds by gas chromatography with MS or MS/MS detection according to methods US EPA 8270D, US EPA 8082A, CSN EN ISO 6468, and US EPA 8000D. Samples were prepared as per CZ_SOP_D06_03_P01 chap. 9.1, 9.4.1.  (LOR^$^ ≡ LoQ^#^)= 3*LOD^£^  **LORs:**   - Naphthalene: 0.03 μg/L; Benzo(a)pyrene: 0.005 μg/L; Phenanthrene: 0.02 μg/L; the rest of the parameters: 0.01 μg/L. - The upper limit depends a lot on the nature of the sample – The lab would roughly estimate it to be around hundreds of mg/L.   **CRM*:** (standards and suppliers)   - CLP Priority Pollutant Internal Standards (CLP PPIS): Absolute Standard - PAH Mix TCL: Sigma-Aldrich - PAH-Mix 9: Dr. Ehrenstorfer - Dibenz(a.h.)antracene-D14: Dr. Ehrenstorfer - Dibenz(a.h.)antracene-D14: Dr. Ehrenstorfer - 2-fluorobiphenyl: Sigma-Aldrich - p-Terphenyl-D14: Sigma-Aldrich | |
| **PHCs** | Determination of extractable compounds in the range of hydrocarbons C10−C40, fractions were calculated from the measured values by gas chromatography with FID detection according to the following methods: CSN EN ISO 9377-2; US EPA 8015; US EPA 3510; TNRCC Method 1006.  (LOR ≡ LoQ)= 3*LOD  The tested measurement range was 10 to 1000 μg/ml of extract, which corresponds, when using a 200ml sample, to a range of 42 to 4170 μg/L sample. In this range, the calibration is linear.  **CRM^*^:** (standards and suppliers)   - two-component standard of diesel with oil, suitable presence of prystan and phytan, e.g. catalogue number BAM-K010; supplier: Chromservis. - standard, which contains mixture of n-alkanes (C_8_-C_40_), e.g. Alkanes-Mix 17, manufacturer: Dr. Ehrenstorfer.   **RM^**^**:   - n-decane (C10), e.g., n-decane; manufacturer: Dr. Ehrenstorfer; supplier Chromservis - n-tetracontane (C40), e.g., n-tetracontane; manufacturer: Dr. Ehrenstorfer; supplier: Chromservis | |
| **BPA and Alkylphenols (APs)** | Determination of alkylphenols and alkylphenol ethoxylates by gas chromatography with MS or MS/MS detection according to method CSN EN ISO 18857-2. Total alkylphenols and alkylphenol ethoxylates were calculated based on the measured values.  (LOR ≡ LoQ)= 3*LOD  **LORs:**   - Nonylphenols: 0.05 μg/L; Octylphenols: 0.01 μg/L; Bisphenol A: 0.05 μg/L. - The upper limit, according to the lab estimations, would be around hundreds of mg/L.   **CRM:** (standards and suppliers)   - Custom mixture of Phenol Ethoxylates: CHIRON AS - 4-n-Nonylphenol D4: Neochema - 4-n-Nonylphenol diethoxylate: Chiron AS - Bisphenol A D16: Sigma-Aldrich | |
| **TOC** | Total organic carbon (TOC) was determined by IR detection according to methods CSN EN 1484 and SM 5310. | |
| **TSS** | Gravimetrical determination of suspended solids according to method SS-EN 872-2:2005. | |
| **Turbidity** | Determined with a 2100Q IS Portable Turbidimeter, HACH (Loveland, CO), calibrated with Formazin primary StablCal Standards. | |
| **Conductivity and temperature** | Determined with pHenomenal® Conductivity/TDS/°C Meter, Handheld, CO 3100 H, VWR (Radnor, PA), calibrated using the control standard KCl solution. | |
| **pH** | Determined with a pH 330i meter, Handheld, WTW GmbH (Weilheim, Germany), calibrated by buffer solutions. | |
| ^*^ Certified Reference Materials (CRM) ^**^ Reference Materials (RM)  ^$^ Limit of Reporting (LOR)  ^#^ Limit of Quantification (LoQ)  ^£^ Limit of Detection (LOD) | | |

Table S2. Characteristics of various Polycyclic Aromatic Hydrocarbons (PAHs)

| PAH | Abbr. | Molecular mass^$^ (Daltons) | No. of rings | fraction | Carcino-genicity group^*^ | Water solubility^¥^ (mg/L at 25˚C) | Vapor pressure^#^ (Pa at 25˚C) | Log K_ow_^$^ | Log K_oc_ (calc.) ^£^ |
| --- | --- | --- | --- | --- | --- | --- | --- | --- | --- |
| **Naphthalene** | Nap | 128 | 2 | LWM | 2B | 31.6 | 10.4 | 3.37 | 2.95 |
| **Acephthylene** | Acyl | 152 | 3 | LWM | − | 16 | 9.0e-1 | 4.00 | 3.13 |
| **Acephthene** | Acen | 154 | 3 | LWM | 3 | 4.5 | 30e-1 | 3.92 | 3.46 |
| **Fluorene** | Flu | 166 | 3 | LWM | 3 | 1.8 | 9.0e-2 | 4.18 | 3.71 |
| **Phenthrene** | Phen | 178 | 3 | LWM | 3 | 1.3 | 2.0e-2 | 4.57 | 3.79 |
| **Anthracene** | Anth | 178 | 3 | LWM | 3 | 0.07 | 1.0e-3 | 4.54 | 4.57 |
| **Fluoranthene** | Flth | 202 | 4 | MWM | 3 | 0.24 | 1.2e-3 | 5.22 | 4.24 |
| **Pyrene** | Pyr | 202 | 4 | MWM | 3 | 0.14 | 6.0e-4 | 5.18 | 4.39 |
| **Benz(a) anthracene** | BaA | 228 | 4 | HWM | 2B | 0.01 | 2.8e-5 | 5.91 | 5.09 |
| **Chrysene** | Chry | 228 | 4 | HWM | 2B | 0.003 | 5.7e-7 | 1.65 | 5.41 |
| **Benzo(b) fluoranthene** | BbF | 252 | 5 | HWM | 2B | <0.001 | − | 5.80 | 5.70 < |
| **Benzo(k) fluoranthene** | BkF | 252 | 5 | HWM | 2B | <0.001 | 5.2e-8 | 6.00 | 5.70 < |
| **Benzo(a)pyrene** | BaP | 252 | 5 | HWM | 1 | <0.001 | 7.0e-7 | 6.04 | 5.70 < |
| **Dibenz(a.h) anthracene** | DahA | 278 | 5 | HWM | 2A | <0.001 | 3.7e-10 | 6.75 | 5.70 < |
| **Benzo(g.h.i) perylene** | Bper | 276 | 6 | HWM | 3 | <0.001 | − | 6.50 | 5.70 < |
| **Indeno(1.2.3.cd) pyrene** | InP | 276 | 6 | HWM | 2B | <0.001 | 6e-8 | 6.58 | 5.70 < |
| ¥ (Monaco et al., 2017)  # (Joa et al., 2009)  $ K_ow_: Octanol-water partition coefficient (Joa et al., 2009)  £ K_oc_: Sediment organic carbon-water partition coefficient (Khodadoust et al., 2005)  * (1): carcinogenic to humans; (2A): probably carcinogenic to humans; (2B): possibly carcinogenic to humans; (3): not classifiable as [carcinogenic](https://www.sciencedirect.com/topics/earth-and-planetary-sciences/carcinogenicity) to humans (IARC Working Group, 2010). | | | | | | | | | |

Table S3. Characteristics of various Petroleum Hydrocarbons (PHCs) (Reed & Stemer, 2002)

| Petroleum Hydrocarbons  (only C10< fractions) | Molecular Weight | Water solubility^¥^ (mg/L at 25˚C) | Vapor pressure^$^ (Pa at 25˚C) | Boiling point (˚C) | Log K_oc_ (calc.) ^£^ |
| --- | --- | --- | --- | --- | --- |
| **Aliphatic** | | | | | |
| C_10_−C_12_ | 160 | 0.026 | 7.9e+1 | 200 | 5.4 |
| C_12_−C_16_ | 200 | 5.9e-4 | 3.5 | 260 | 6.7 |
| C_16_−C_21_ | 270 | 1.0e-6 | 1.7e-1 | 320 | 8.8 |
| **Aromatic** | | | | | |
| C_10_−C_12_ | 130 | 25 | 7.8e+1 | 200 | 3.4 |
| C_12_−C_16_ | 150 | 5.8 | 3.5 | 260 | 3.7 |
| C_16_−C_21_ | 190 | 0.51 | 1.7e-1 | 320 | 4.2 |
| C_21_−C_35_ | 240 | 0.0066 | 7.9e-4 | 340 | 5.1 |
| Note: Values are based on pure substances; behaviour may differ in complex mixtures.  £ K_oc_: Sediment organic carbon-water partition coefficient | | | | | |

| **(a)** 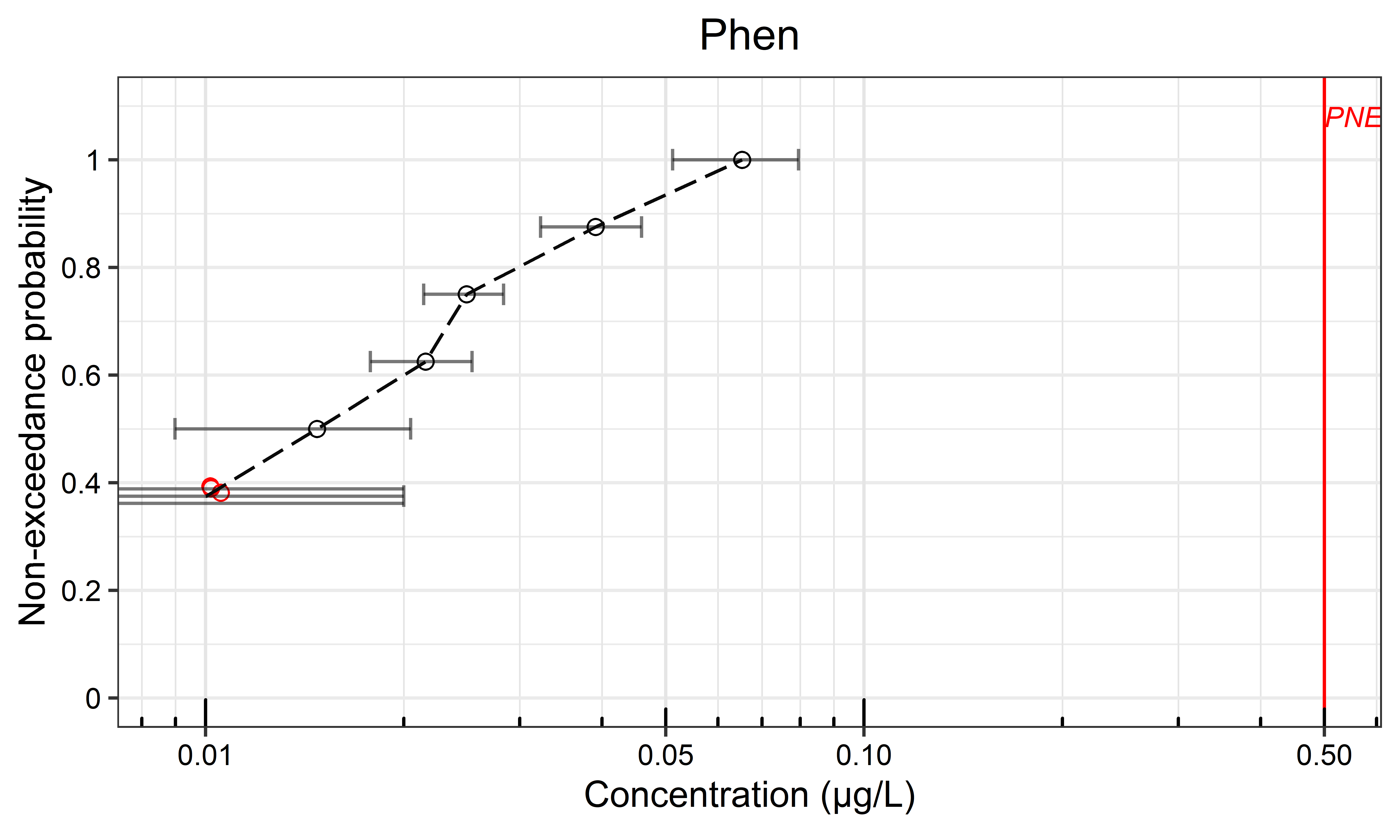 | **(b)** 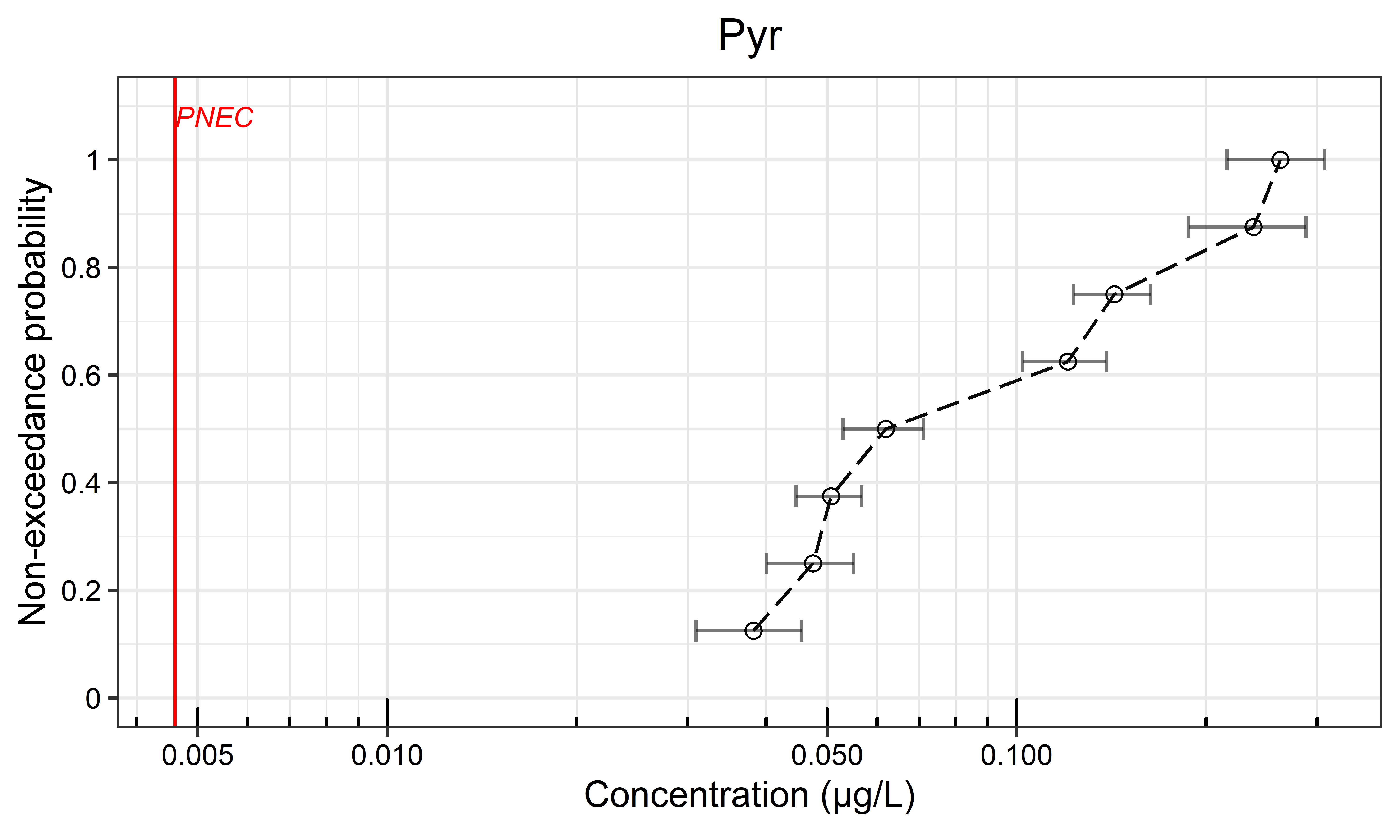 | **(c)** 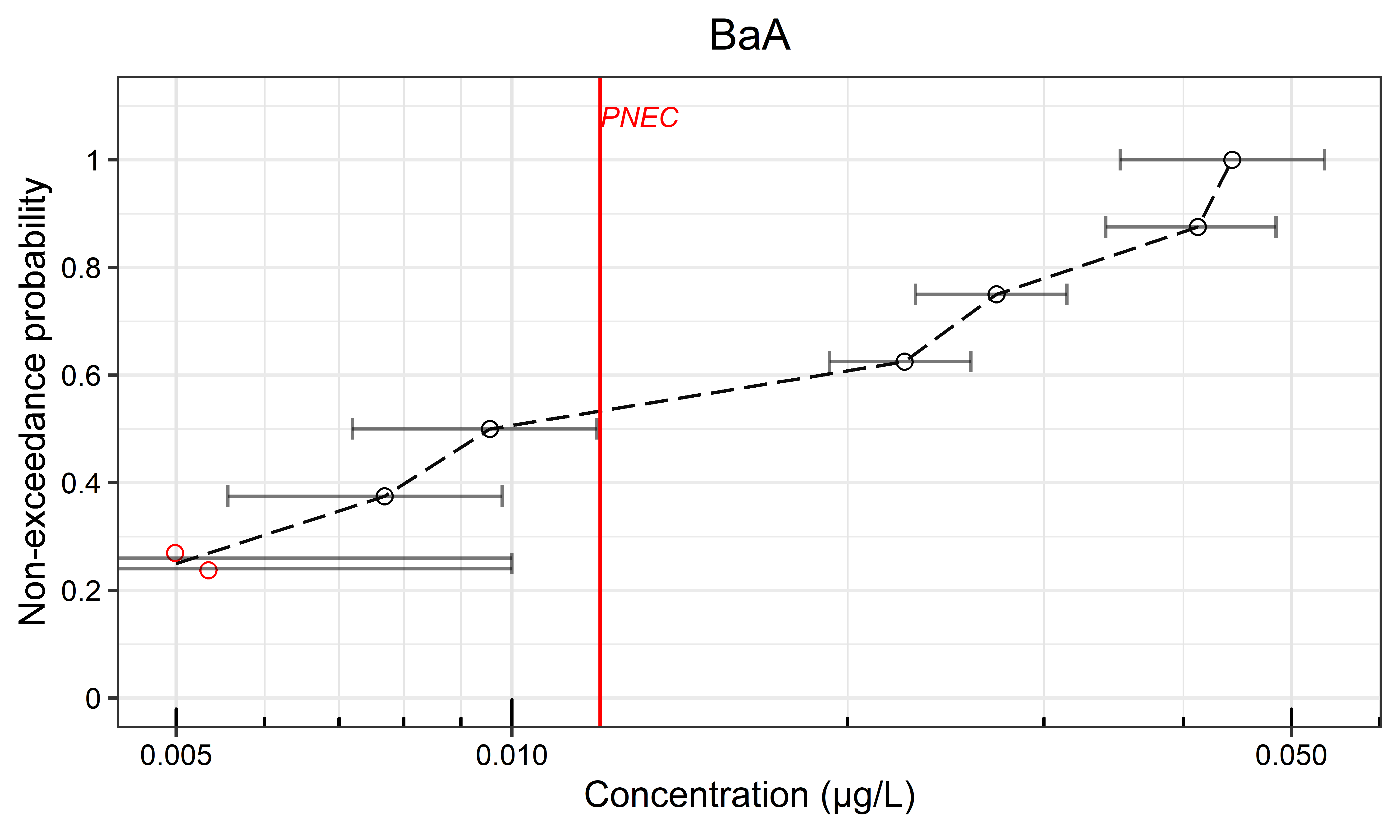 |
| --- | --- | --- |
| **(d)** 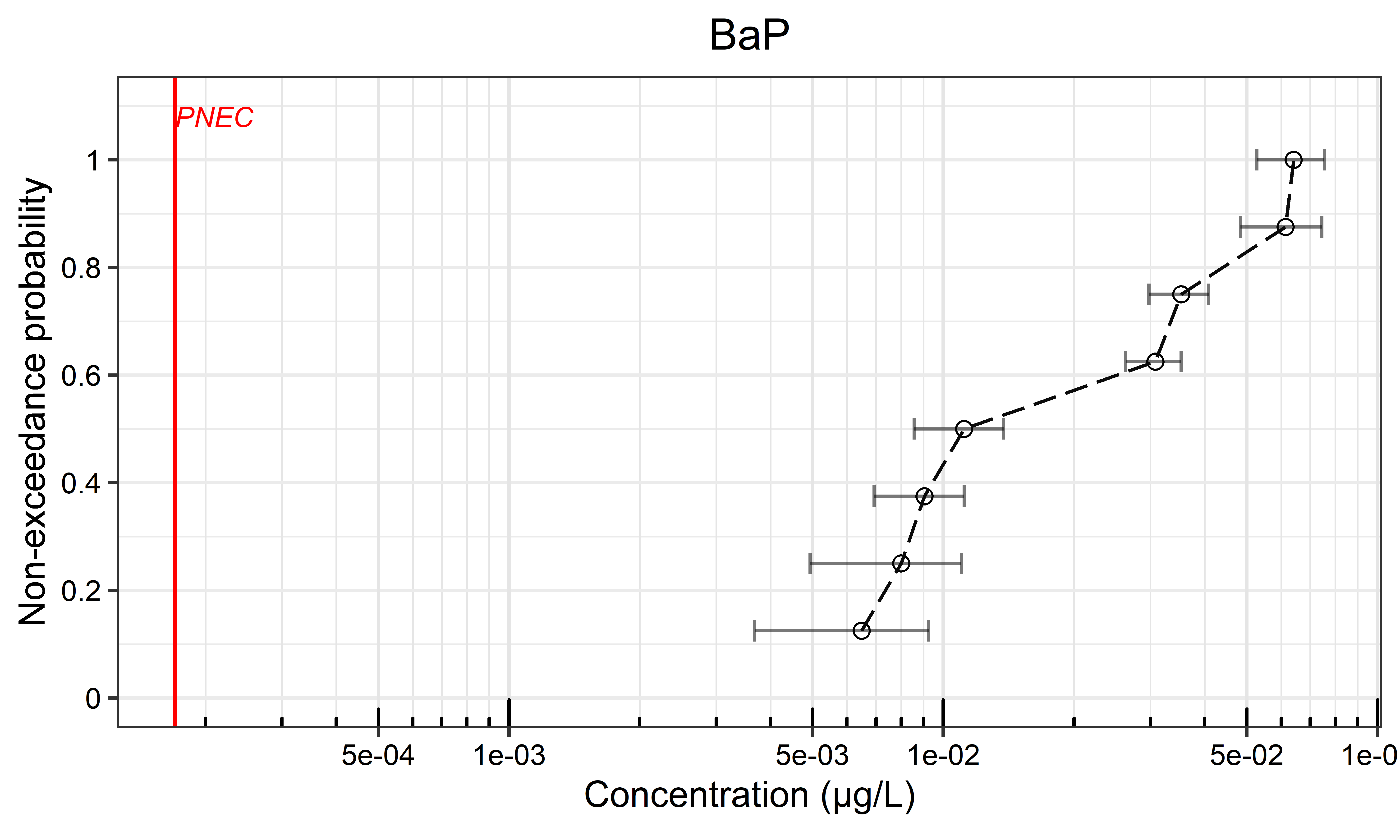 | **(e)** 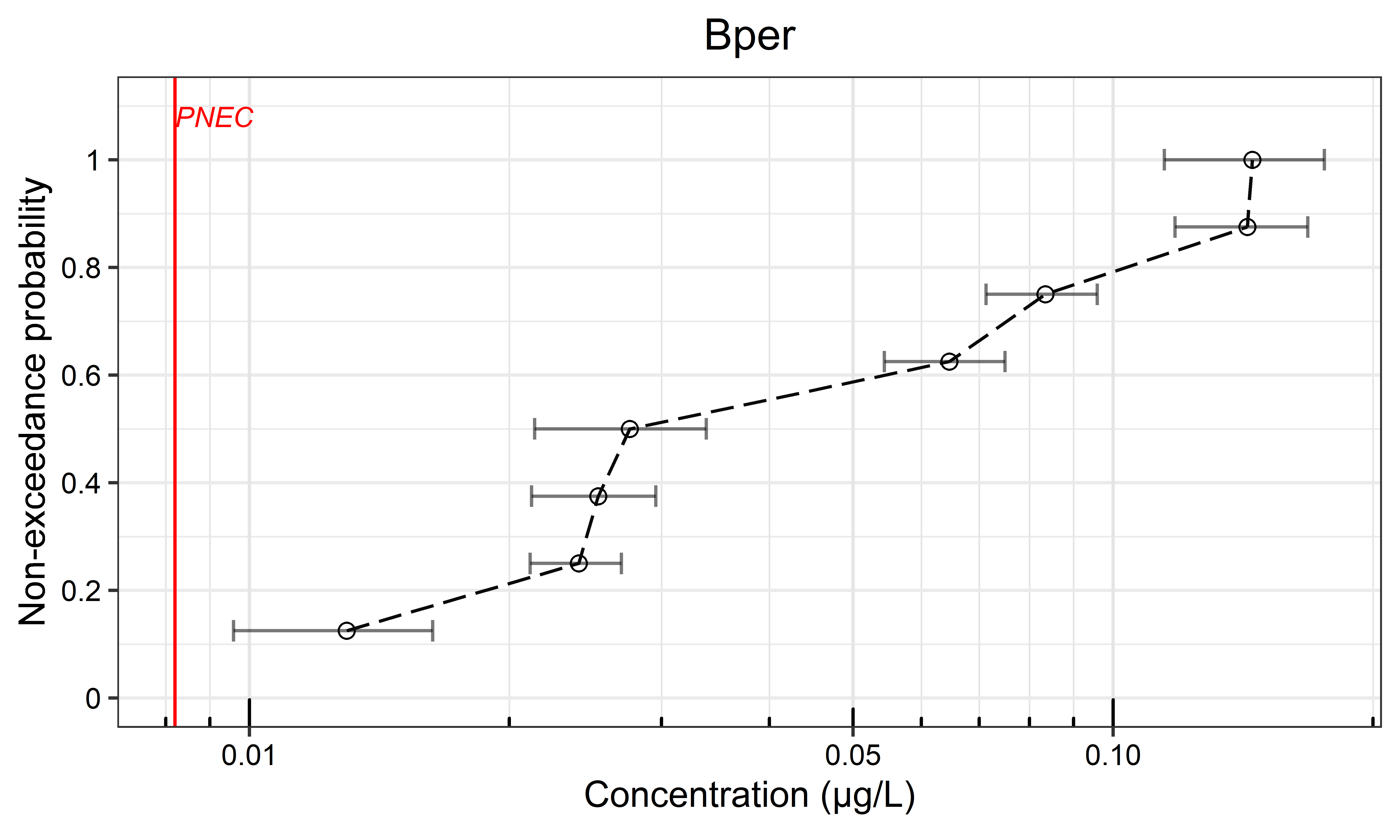 | **(f)** **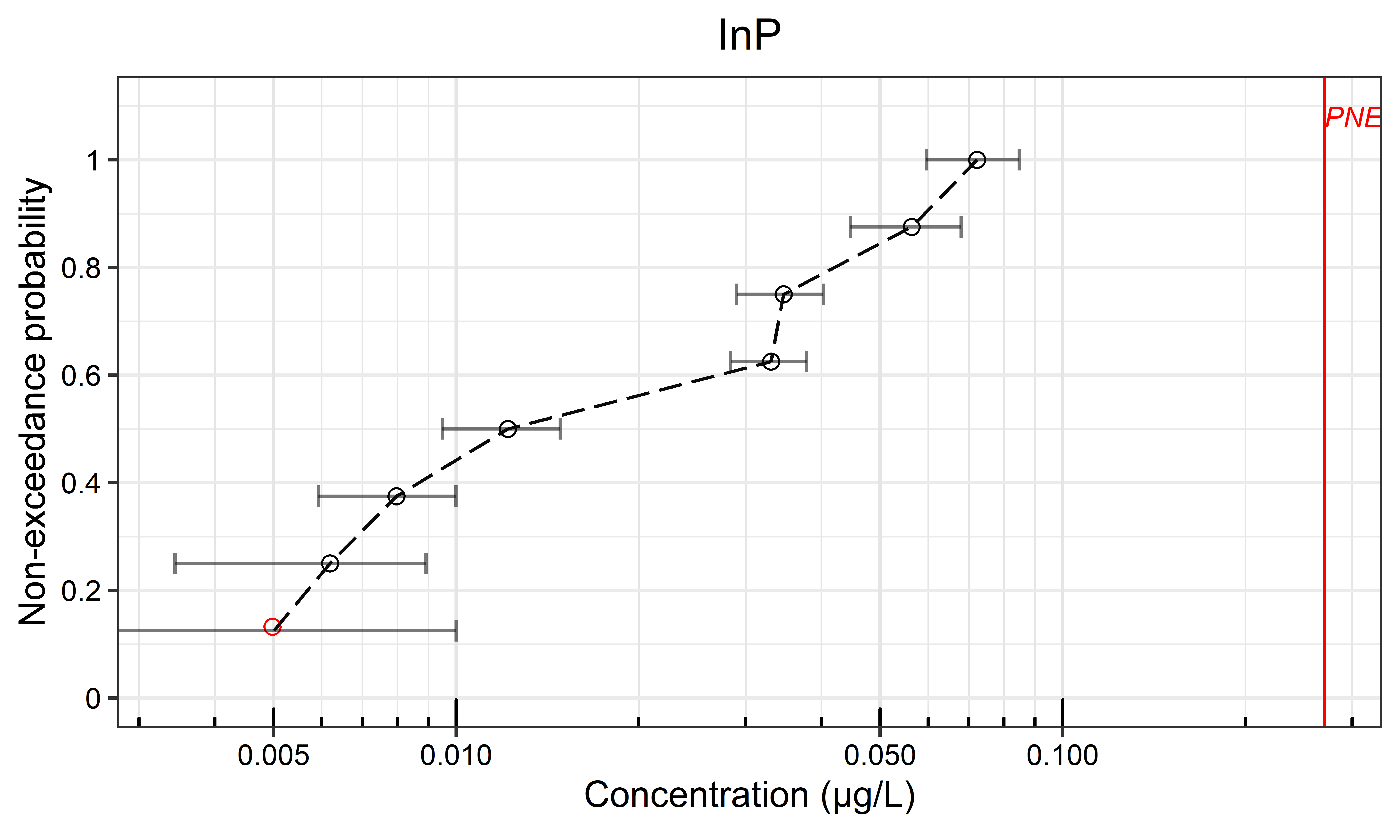** |
| **(g) 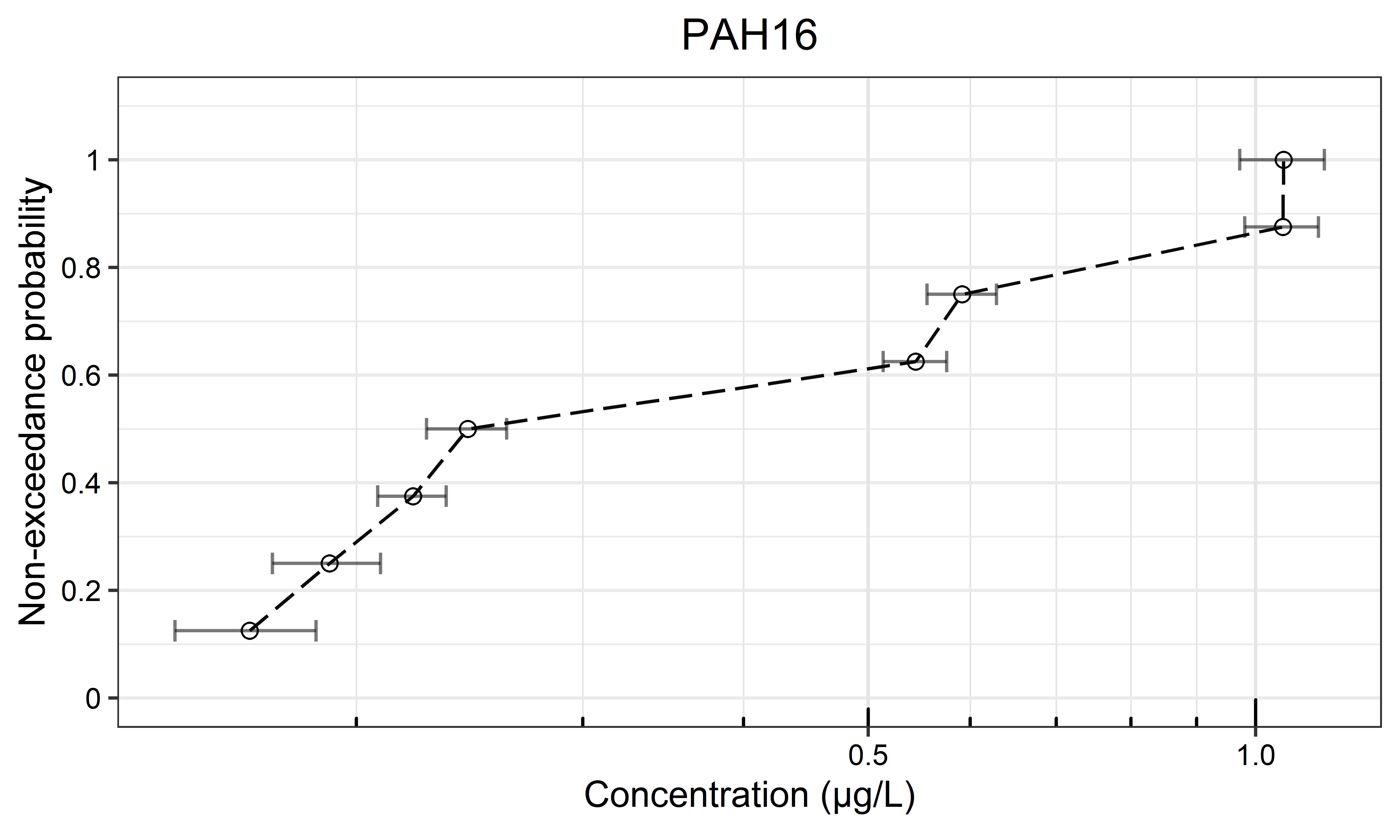** | **(h) 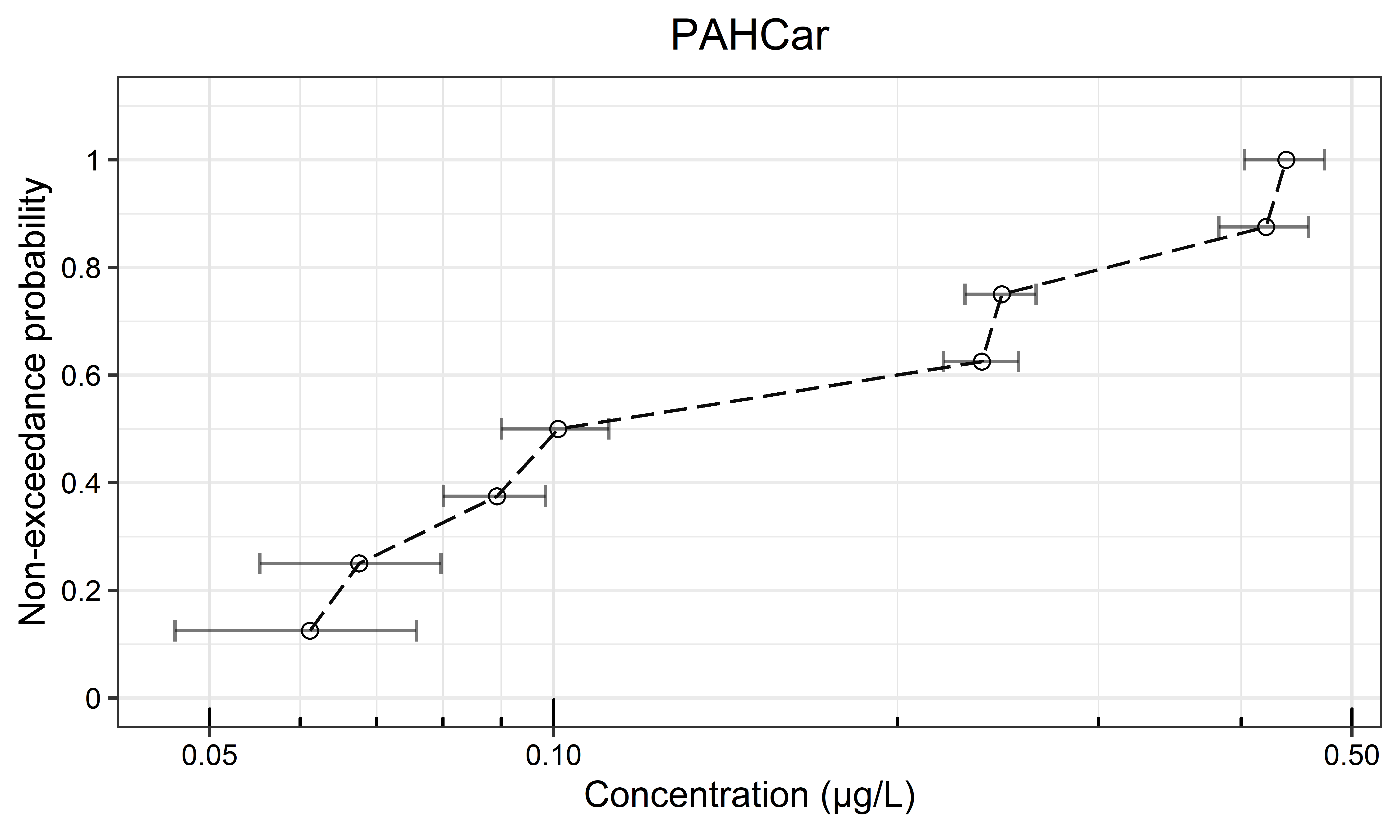** | **(i) 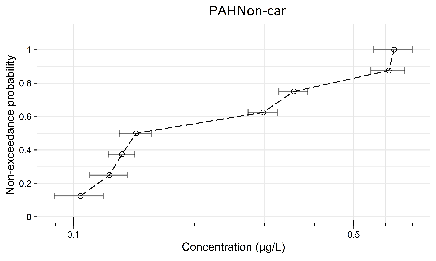** |
| **(j)** **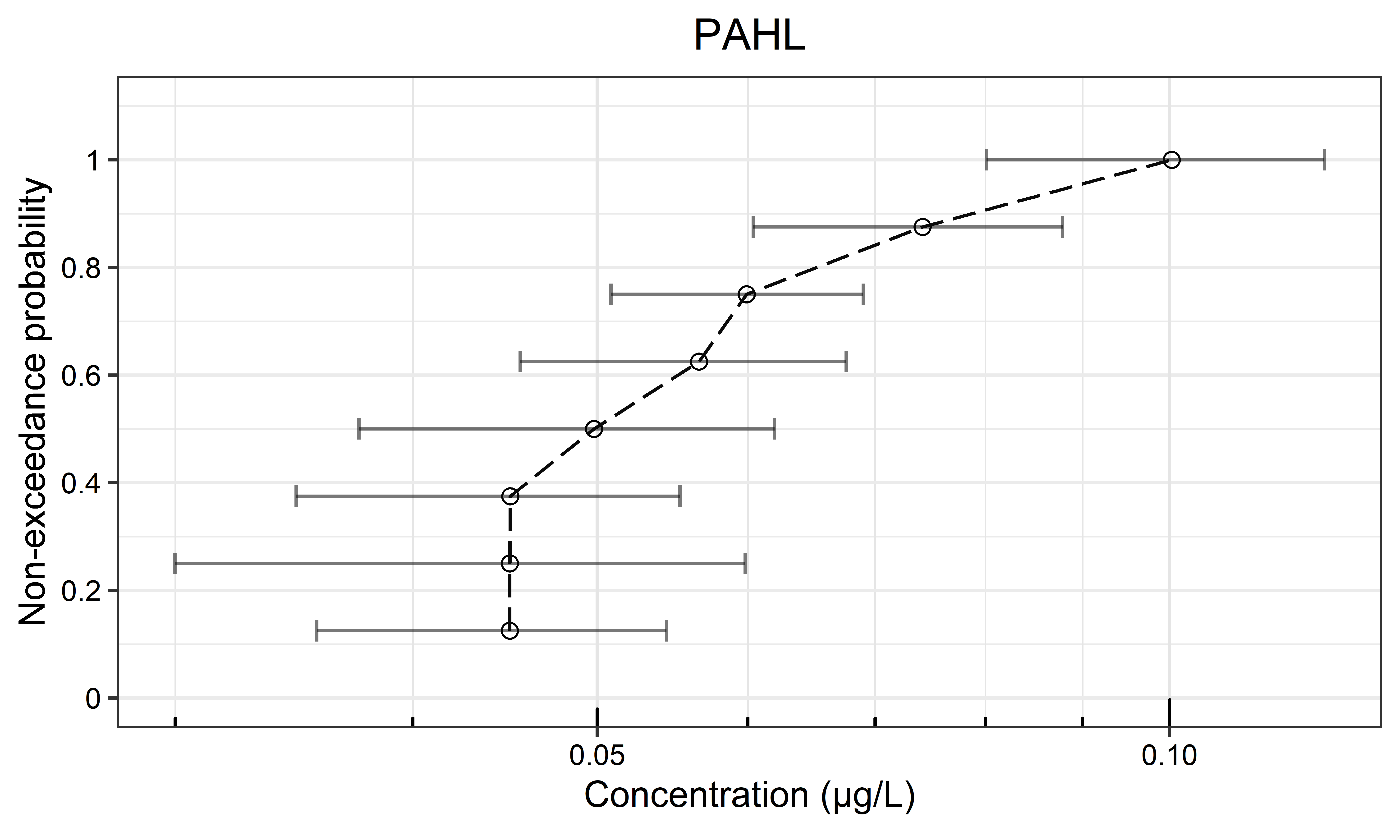** | **(k) 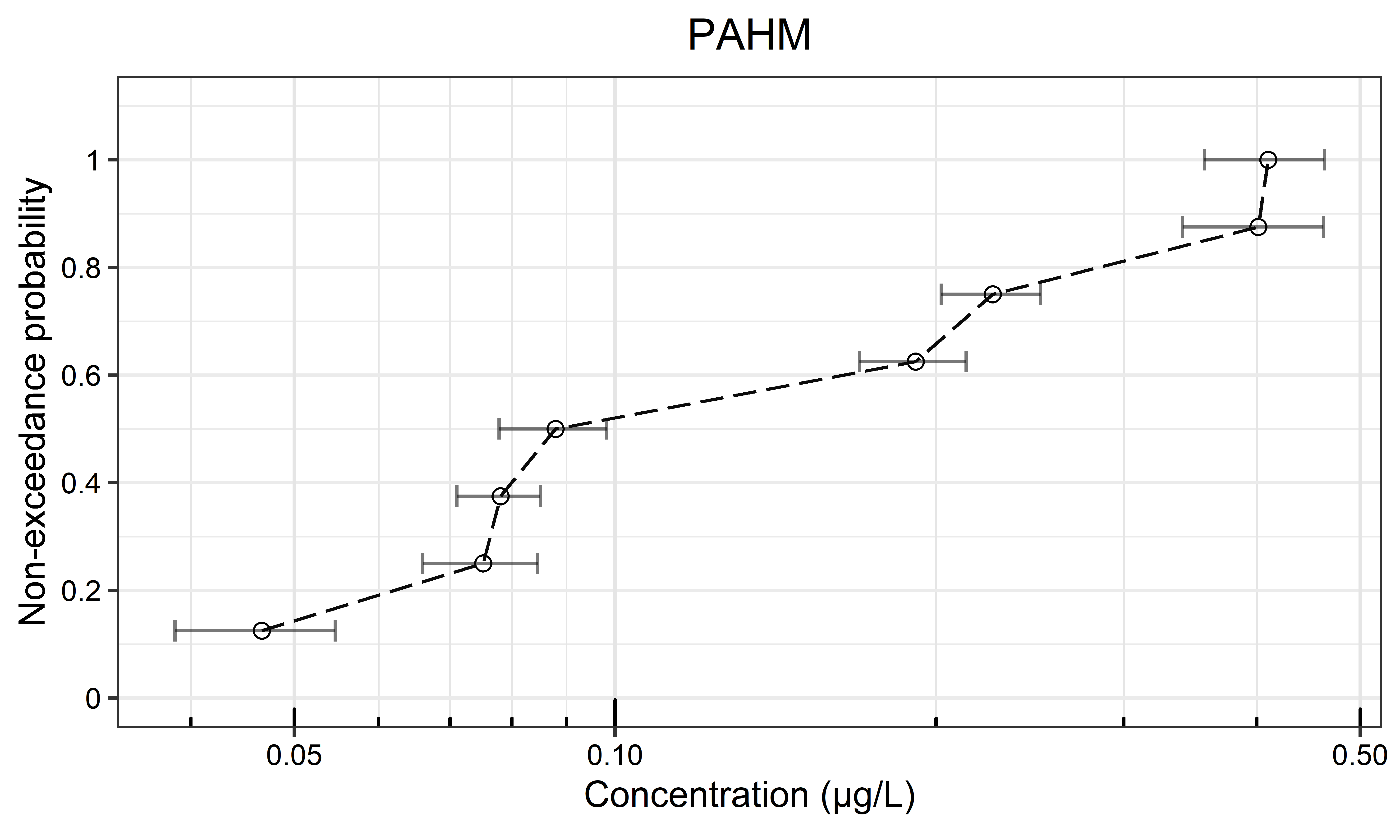** | **(l) 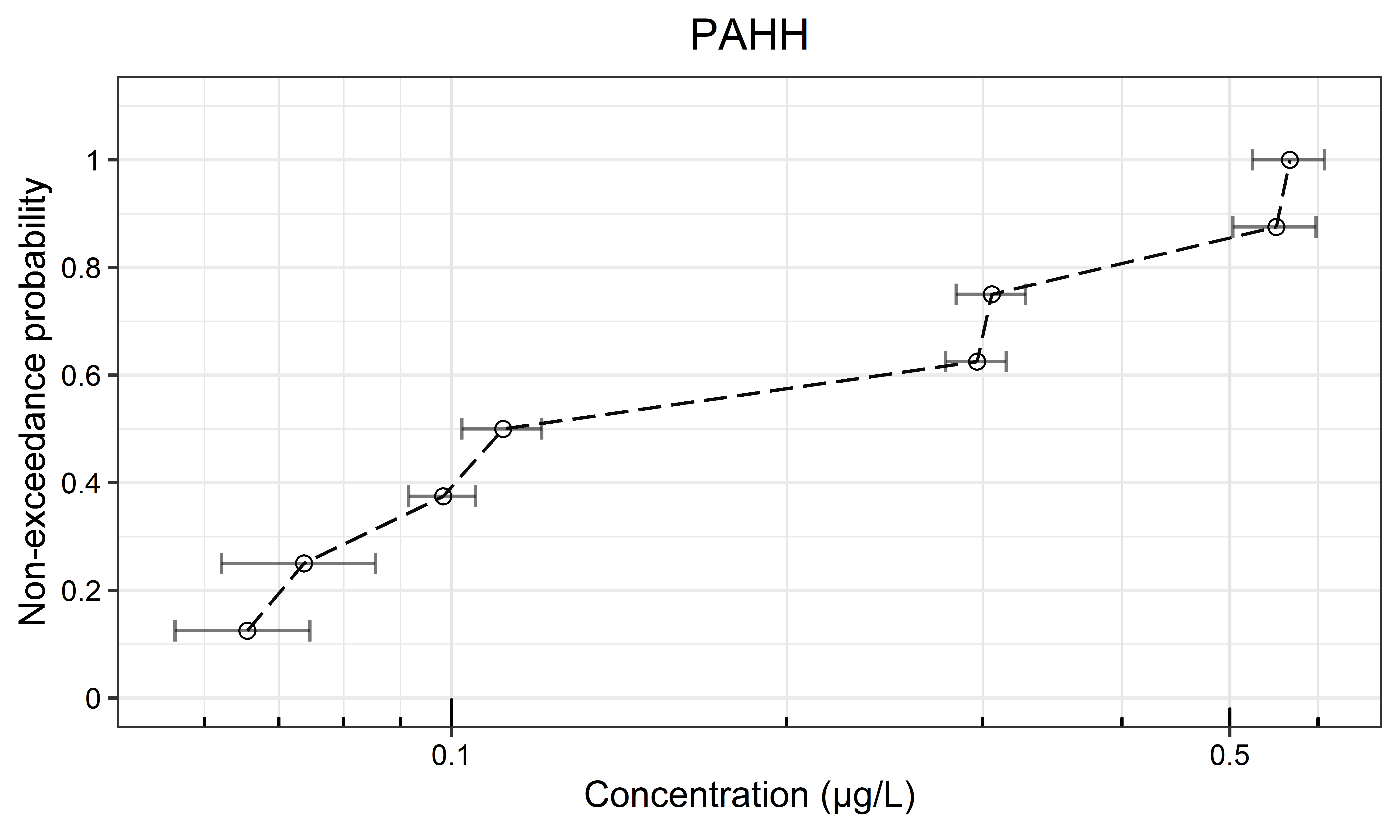** |
| **(m) 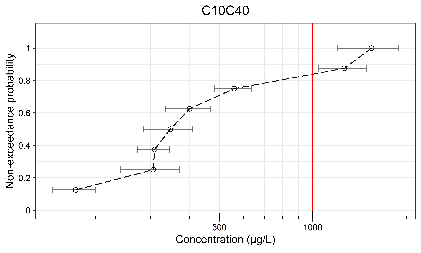** | **(n) 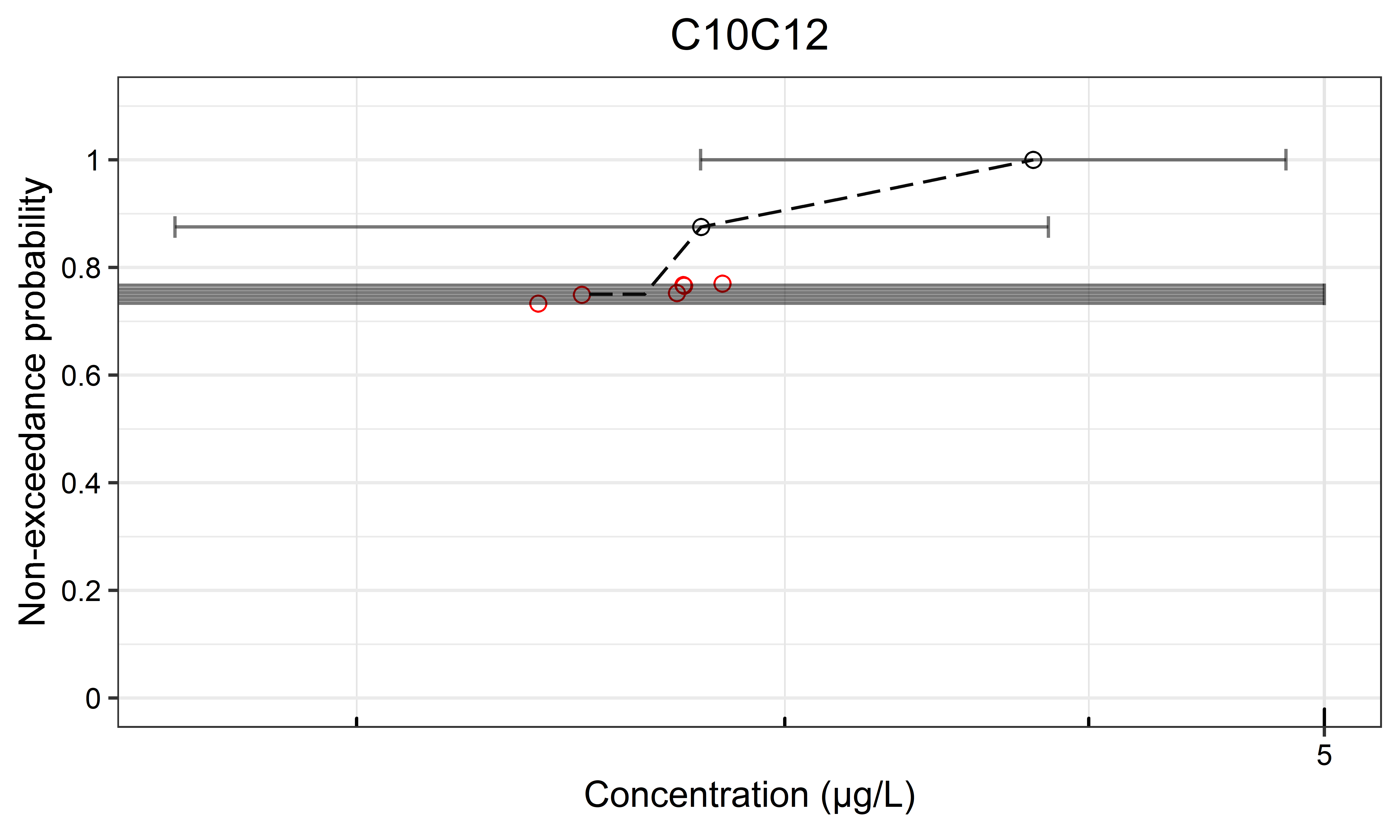** | **(o) 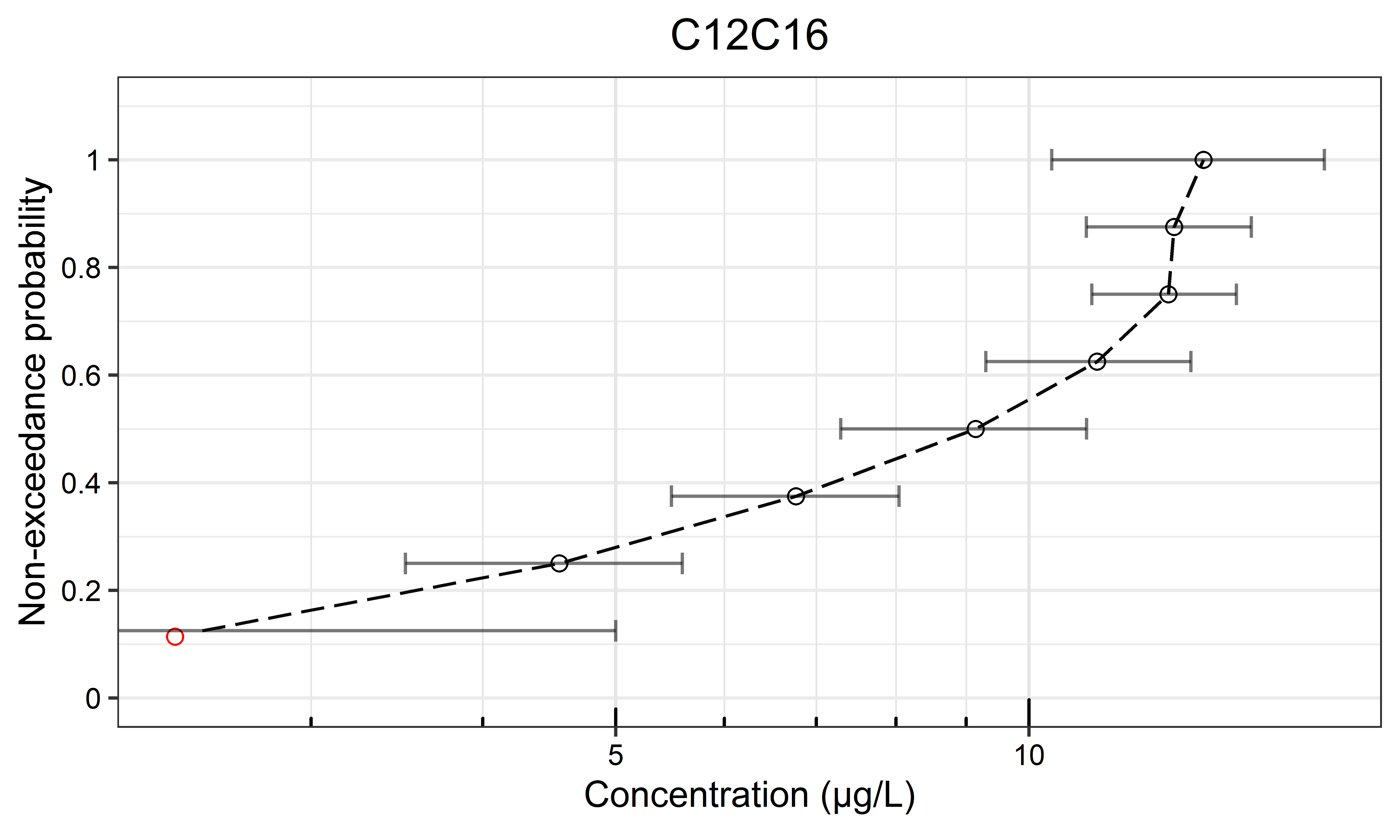** |
| **(p) 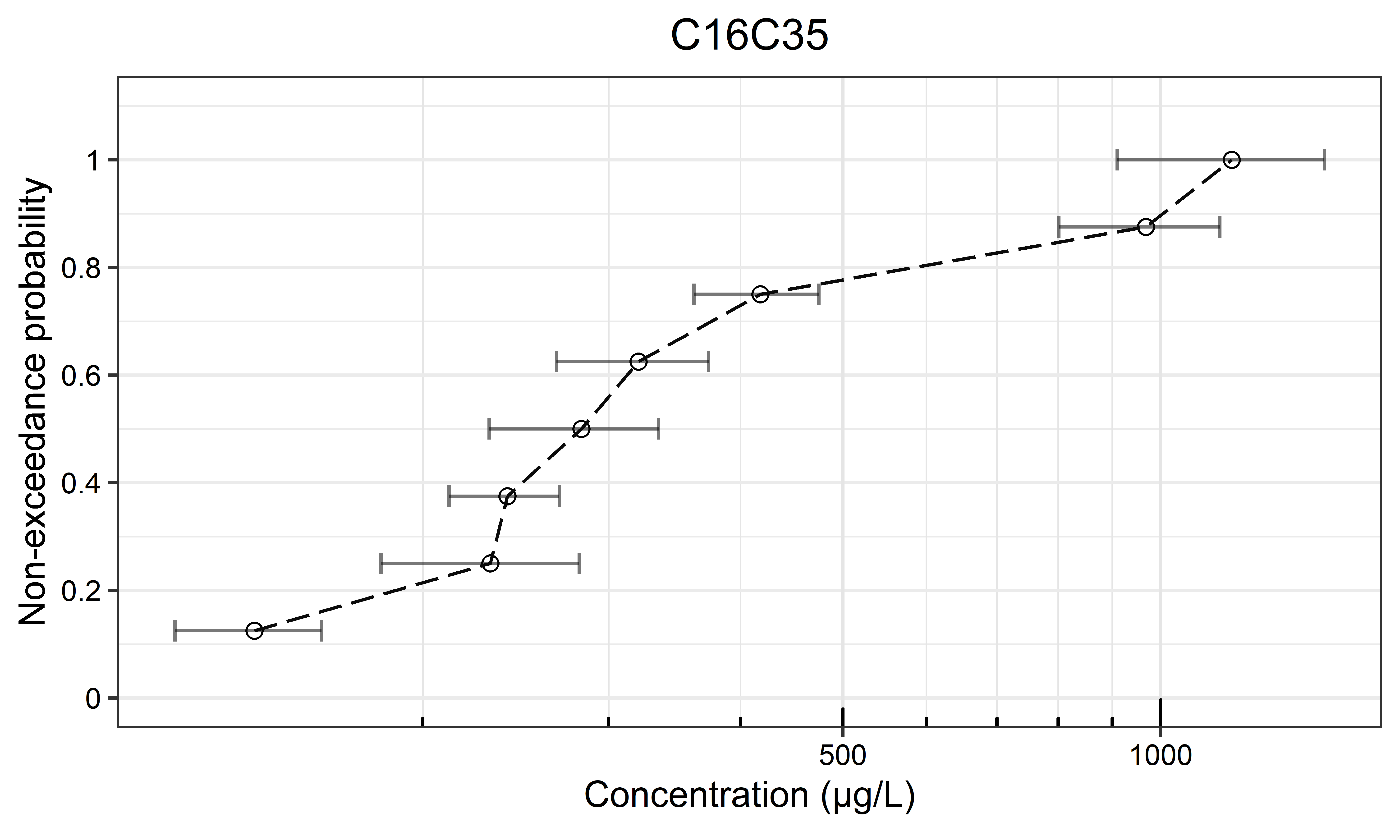** | **(q) 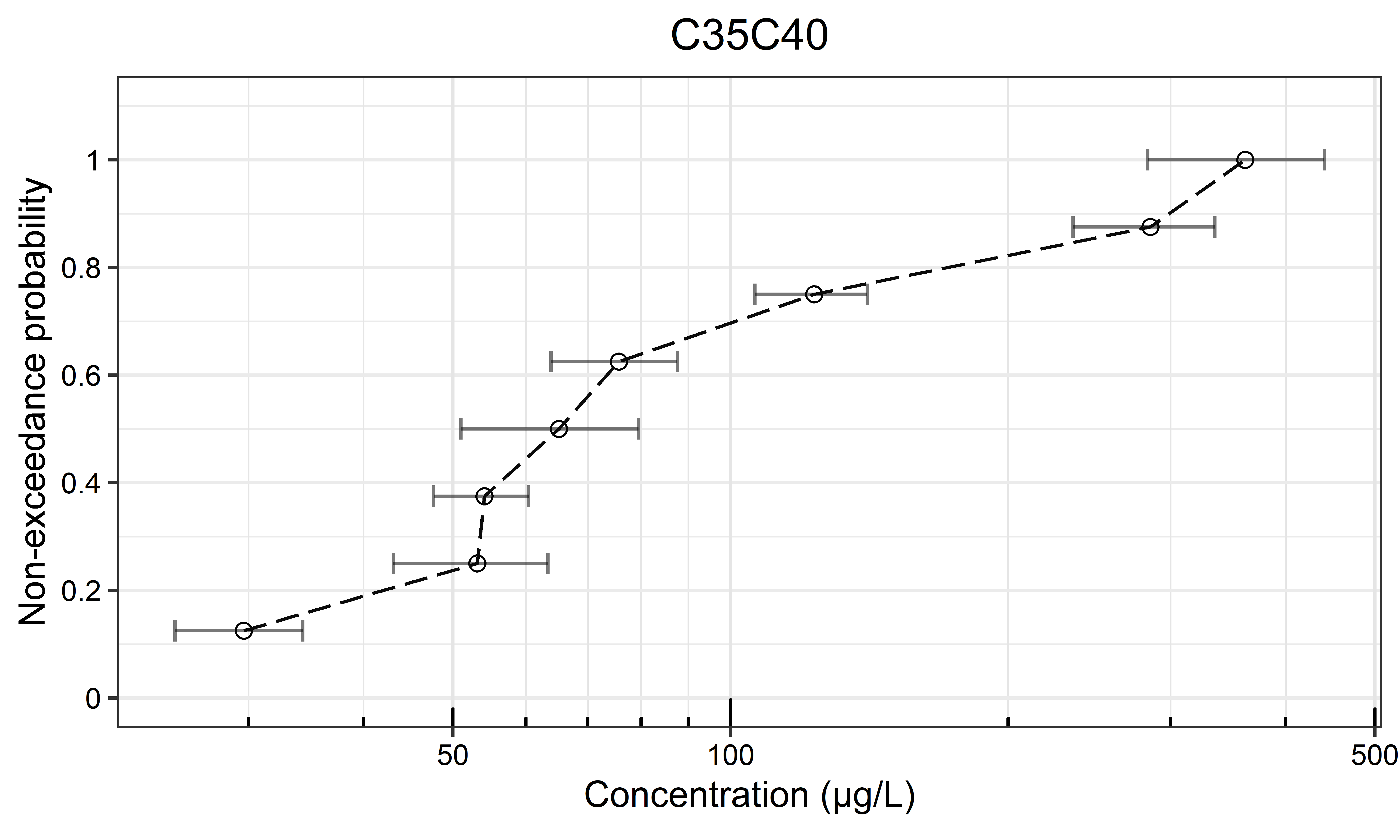** | **(r) 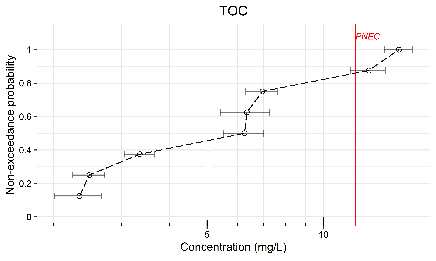** |
| **(s) 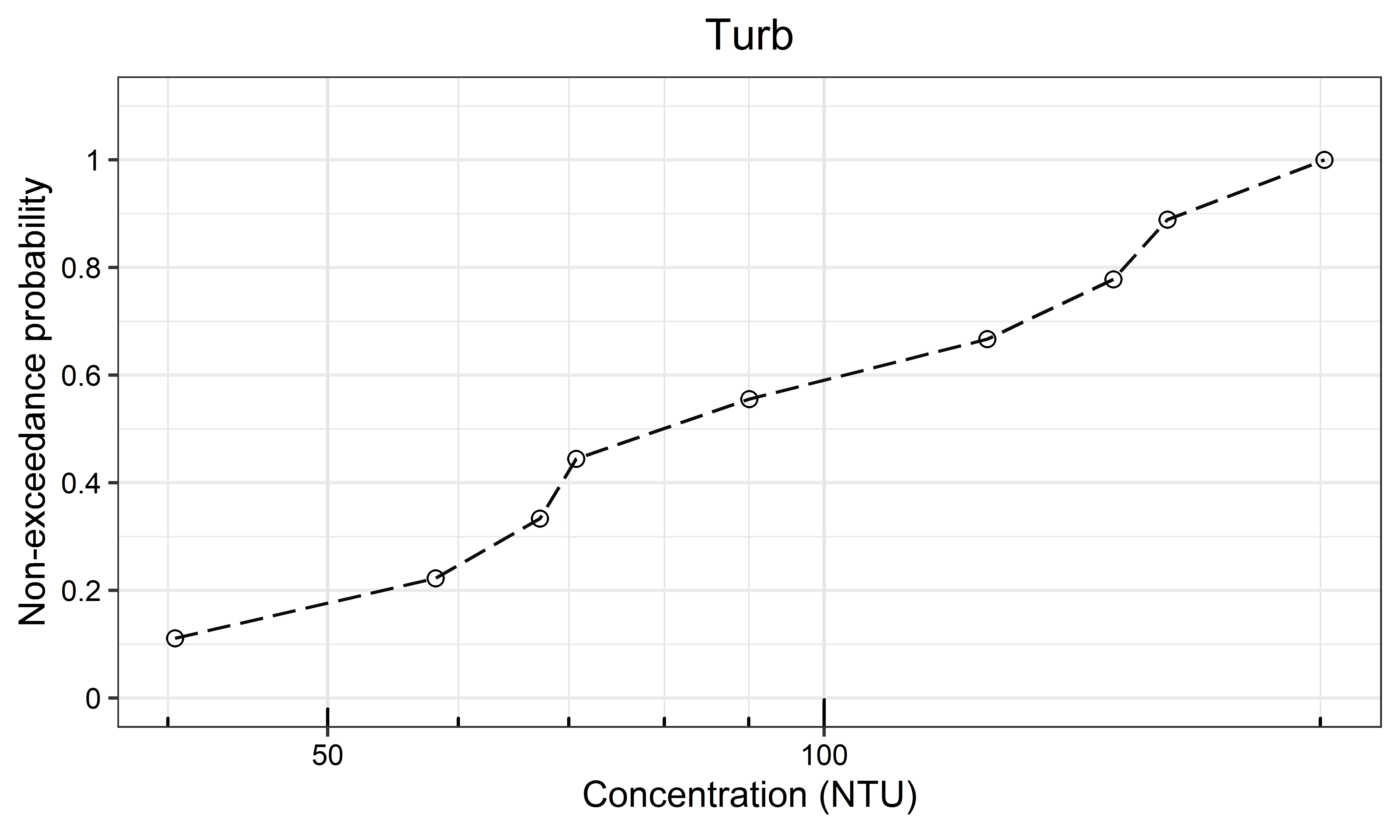** | **(t) 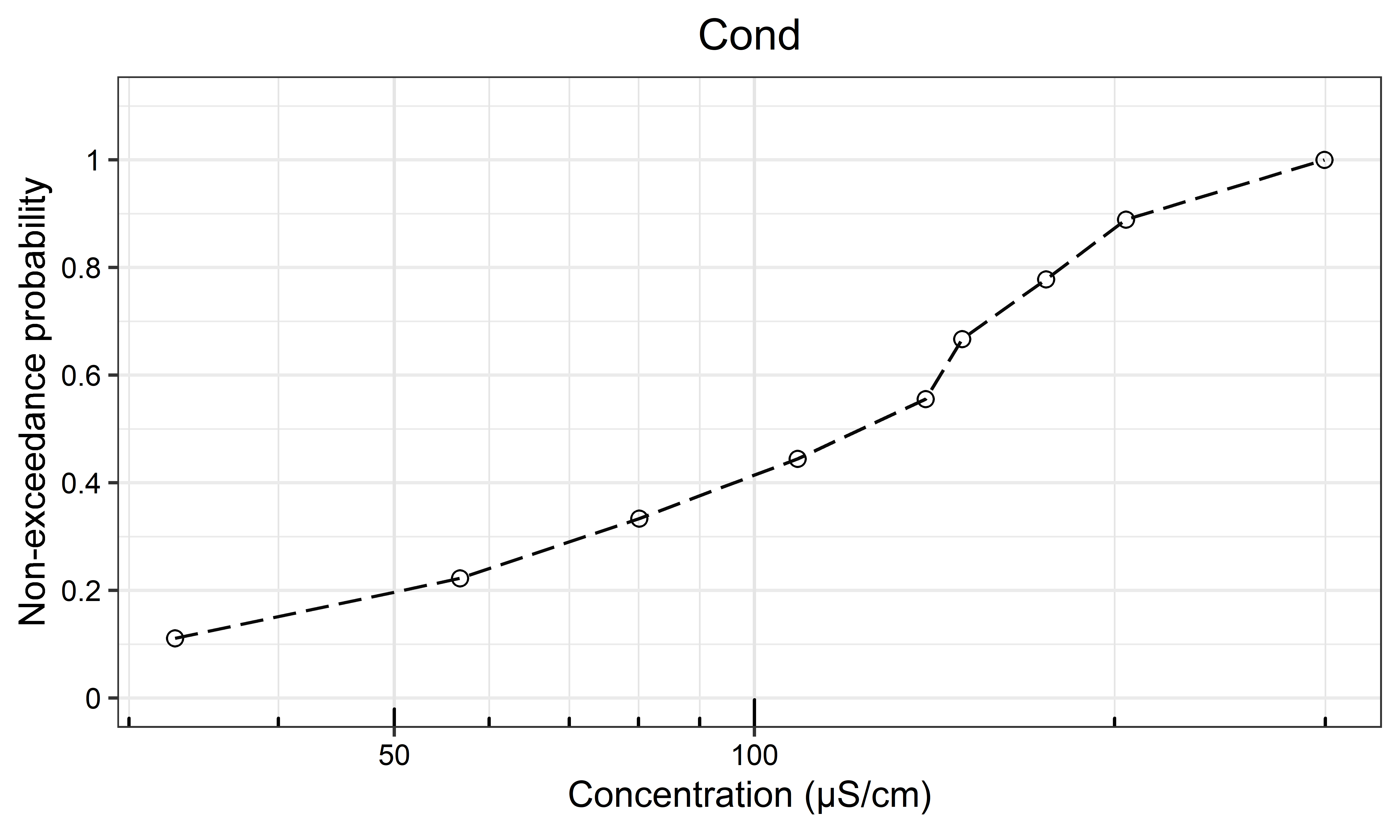** |  |

Figure S2. Non-exceedance probability (NEP) plots based on the estimated EMC values for each OMP (Black points are detects and red points censored data (non-detects); Error bars show EMC errors (uncertainties); Red lines represent lowest PNEC levels for freshwater based on water quality objectives (WQOs))

*Table S4. Correlation coefficients matrix for all parameters (Kendall’s Tau ranks are underlined while Spearman’s correlation ranks are not) (Dark green: very strong; Green: strong; Yellow: moderate; Orange: weak; Red: very weak). The values shown in black are statistically significant (p-value < 0.05), while those in grey are not.*

| **Phen** | **Flth** | **Pyr** | **BaA** | **Chry** | **BbF** | **BkF** | **BaP** | **DahA** | **Bper** | **InP** | **PAH16** | **PAHCar** | **PAHNon-car** | **PAHL** | **PAHM** | **PAHH** | **OP** | **NP** | **BPA** | **C_10_-C_40_** | **C_10_-C_12_** | **C12-C_16_** | **C_16_-C_35_** | **C_35_-C_40_** | **TOC** | **TSS** | **Turb** | **Cond** | **pH** | **Depth** | **ADP** | **I_mean_** | **I_peak_** |  |
| --- | --- | --- | --- | --- | --- | --- | --- | --- | --- | --- | --- | --- | --- | --- | --- | --- | --- | --- | --- | --- | --- | --- | --- | --- | --- | --- | --- | --- | --- | --- | --- | --- | --- | --- |
| 0.79 | 0.79 | 0.71 | 0.71 | 0.71 | 0.71 | 0.64 | 0.64 | 0.71 | 0.79 | 0.64 | 0.79 | 0.64 | 0.79 | 0.79 | 0.71 | 0.71 | 0.50 | -0.11 | -0.14 | 0.79 | 0.04 | 0.39 | 0.79 | 0.79 | -0.21 | 0.57 | 0.80 | 0.40 | 0.00 | -0.14 | -0.07 | 0.21 | -0.07 | **Phen** |
|  | 1.00 | 0.88 | 0.68 | 0.75 | 0.88 | 0.61 | 0.79 | 0.71 | 0.76 | 0.57 | 0.91 | 0.86 | 0.91 | 0.93 | 0.88 | 0.81 | 0.46 | -0.04 | -0.17 | 0.83 | 0.04 | 0.39 | 0.83 | 0.79 | -0.29 | 0.88 | 0.83 | 0.60 | -0.20 | -0.14 | -0.14 | 0.31 | -0.10 | **Flth** |
|  |  | 1.00 | 0.68 | 0.75 | 1.00 | 0.75 | 0.95 | 0.71 | 0.83 | 0.86 | 0.98 | 0.98 | 0.98 | 0.86 | 1.00 | 0.98 | 0.61 | 0.04 | -0.29 | 0.95 | 0.04 | 0.39 | 0.95 | 0.81 | -0.26 | 0.76 | 0.94 | 0.37 | 0.14 | 0.10 | -0.14 | 0.38 | 0.10 | **Pyr** |
|  |  |  | 0.82 | 0.75 | 0.68 | 0.75 | 0.75 | 0.62 | 0.75 | 0.75 | 0.75 | 0.75 | 0.75 | 0.75 | 0.68 | 0.68 | 0.61 | -0.14 | -0.18 | 0.75 | 0.04 | 0.29 | 0.75 | 0.68 | -0.25 | 0.46 | 0.73 | 0.33 | 0.20 | -0.11 | -0.11 | 0.25 | -0.18 | **BaA** |
|  |  |  |  | 0.96 | 0.75 | 0.68 | 0.82 | 0.62 | 0.68 | 0.79 | 0.82 | 0.82 | 0.82 | 0.61 | 0.75 | 0.75 | 0.68 | 0.04 | -0.39 | 0.82 | 0.04 | 0.50 | 0.82 | 0.75 | -0.39 | 0.46 | 0.77 | 0.14 | 0.09 | 0.11 | -0.25 | 0.32 | 0.04 | **Chry** |
|  |  |  |  |  | 1.00 | 0.75 | 0.95 | 0.71 | 0.83 | 0.86 | 0.98 | 0.98 | 0.98 | 0.86 | 1.00 | 0.98 | 0.61 | 0.04 | -0.29 | 0.95 | 0.04 | 0.39 | 0.95 | 0.81 | -0.26 | 0.76 | 0.94 | 0.37 | 0.14 | 0.10 | -0.14 | 0.38 | 0.09 | **BbF** |
|  |  |  |  |  |  | 0.82 | 0.82 | 0.62 | 0.68 | 0.82 | 0.68 | 0.82 | 0.68 | 0.68 | 0.75 | 0.75 | 0.54 | -0.07 | -0.11 | 0.68 | 0.04 | 0.21 | 0.68 | 0.61 | -0.18 | 0.54 | 0.73 | 0.33 | 0.20 | -0.04 | -0.04 | 0.32 | -0.11 | **BkF** |
|  |  |  |  |  |  |  | 1.00 | 0.62 | 0.88 | 0.93 | 0.93 | 0.98 | 0.93 | 0.79 | 0.95 | 0.98 | 0.68 | 0.04 | -0.45 | 0.95 | 0.04 | 0.39 | 0.95 | 0.86 | -0.43 | 0.64 | 0.89 | 0.26 | 0.31 | 0.17 | -0.21 | 0.45 | 0.10 | **BaP** |
|  |  |  |  |  |  |  |  | 0.71 | 0.71 | 0.62 | 0.71 | 0.62 | 0.71 | 0.71 | 0.71 | 0.71 | 0.33 | -0.10 | -0.05 | 0.71 | 0.05 | 0.38 | 0.71 | 0.71 | -0.14 | 0.62 | 0.70 | 0.10 | -0.30 | -0.05 | 0.14 | 0.05 | -0.05 | **DahA*** |
|  |  |  |  |  |  |  |  |  | 1.00 | 0.71 | 0.86 | 0.81 | 0.86 | 0.83 | 0.83 | 0.91 | 0.54 | -0.18 | -0.21 | 0.93 | 0.04 | 0.39 | 0.93 | 0.98 | -0.33 | 0.69 | 0.94 | 0.37 | 0.14 | -0.07 | -0.21 | 0.31 | -0.12 | **Bper** |
|  |  |  |  |  |  |  |  |  |  | 0.93 | 0.79 | 0.93 | 0.79 | 0.64 | 0.86 | 0.86 | 0.64 | 0.00 | -0.21 | 0.79 | 0.04 | 0.32 | 0.79 | 0.64 | -0.21 | 0.43 | 0.89 | 0.26 | 0.31 | 0.07 | -0.07 | 0.29 | -0.00 | **InP** |
|  |  |  |  |  |  |  |  |  |  |  | 1.00 | 0.95 | 1.00 | 0.88 | 0.98 | 0.95 | 0.68 | -0.04 | -0.31 | 0.98 | 0.04 | 0.46 | 0.98 | 0.83 | -0.33 | 0.74 | 0.94 | 0.37 | 0.14 | 0.05 | -0.19 | 0.26 | -0.00 | **PAH16** |
|  |  |  |  |  |  |  |  |  |  |  |  | 1.00 | 0.95 | 0.83 | 0.98 | 0.95 | 0.68 | 0.04 | -0.41 | 0.93 | 0.04 | 0.32 | 0.93 | 0.79 | -0.38 | 0.69 | 0.89 | 0.26 | 0.31 | 0.12 | -0.17 | 0.41 | 0.07 | **PAHCar** |
|  |  |  |  |  |  |  |  |  |  |  |  |  | 1.00 | 0.88 | 0.98 | 0.95 | 0.68 | -0.04 | -0.31 | 0.98 | 0.04 | 0.46 | 0.98 | 0.83 | -0.33 | 0.74 | 0.94 | 0.37 | 0.14 | 0.05 | -0.19 | 0.26 | -0.00 | **PAHNon-car** |
|  |  |  |  |  |  |  |  |  |  |  |  |  |  | 1.00 | 0.86 | 0.81 | 0.46 | -0.18 | 0.05 | 0.83 | 0.04 | 0.25 | 0.83 | 0.79 | -0.12 | 0.81 | 0.94 | 0.71 | 0.03 | -0.33 | -0.02 | 0.10 | -0.32 | **PAHL** |
|  |  |  |  |  |  |  |  |  |  |  |  |  |  |  | 1.00 | 0.98 | 0.61 | 0.04 | -0.29 | 0.95 | 0.04 | 0.39 | 0.95 | 0.81 | -0.26 | 0.76 | 0.94 | 0.37 | 0.14 | 0.10 | -0.14 | 0.38 | 0.10 | **PAHM** |
|  |  |  |  |  |  |  |  |  |  |  |  |  |  |  |  | 1.00 | 0.61 | 0.04 | -0.33 | 0.98 | 0.04 | 0.46 | 0.98 | 0.88 | -0.31 | 0.71 | 0.94 | 0.37 | 0.14 | 0.14 | -0.19 | 0.43 | 0.12 | **PAHH** |
|  |  |  |  |  |  |  |  |  |  |  |  |  |  |  |  |  | 0.89 | -0.04 | -0.46 | 0.68 | -0.04 | 0.21 | 0.68 | 0.46 | -0.54 | 0.18 | 0.40 | 0.00 | 0.53 | -0.04 | -0.39 | 0.18 | -0.11 | **OP** |
|  |  |  |  |  |  |  |  |  |  |  |  |  |  |  |  |  |  | 0.54 | -0.39 | -0.04 | 0.00 | 0.04 | -0.04 | -0.11 | -0.18 | -0.04 | -0.20 | -0.33 | -0.47 | 0.46 | 0.04 | 0.25 | 0.54 | **NP** |
|  |  |  |  |  |  |  |  |  |  |  |  |  |  |  |  |  |  |  | 1.00 | -0.36 | 0.04 | -0.25 | -0.36 | -0.31 | 0.88 | 0.05 | 0.14 | 0.66 | -0.09 | -0.60 | 0.52 | -0.52 | -0.38 | **BPA** |
|  |  |  |  |  |  |  |  |  |  |  |  |  |  |  |  |  |  |  |  | 1.00 | 0.04 | 0.54 | 1.00 | 0.91 | -0.38 | 0.69 | 0.94 | 0.37 | 0.14 | 0.10 | -0.24 | 0.31 | 0.02 | **C_10_-C_40_** |
|  |  |  |  |  |  |  |  |  |  |  |  |  |  |  |  |  |  |  |  |  | 0.04 | 0.04 | 0.04 | 0.04 | 0.04 | 0.04 | 0.07 | 0.07 | -0.07 | -0.04 | 0.04 | -0.04 | -0.04 | **C_10_-C_12_** |
|  |  |  |  |  |  |  |  |  |  |  |  |  |  |  |  |  |  |  |  |  |  | 0.96 | 0.54 | 0.46 | -0.25 | 0.25 | 0.13 | 0.00 | -0.40 | 0.11 | -0.46 | 0.04 | 0.18 | **C_12_-C_16_** |
|  |  |  |  |  |  |  |  |  |  |  |  |  |  |  |  |  |  |  |  |  |  |  | 1.00 | 0.91 | -0.38 | 0.69 | 0.94 | 0.37 | 0.14 | 0.10 | -0.24 | 0.31 | 0.02 | **C_16_-C_35_** |
|  |  |  |  |  |  |  |  |  |  |  |  |  |  |  |  |  |  |  |  |  |  |  |  | 1.00 | -0.43 | 0.74 | 0.83 | 0.26 | -0.09 | 0.00 | -0.29 | 0.45 | -0.02 | **C_35_-C_40_** |
|  |  |  |  |  |  |  |  |  |  |  |  |  |  |  |  |  |  |  |  |  |  |  |  |  | 1.00 | 0.19 | 0.33 | 0.78 | -0.13 | -0.52 | 0.66 | -0.17 | -0.28 | **TOC** |
|  |  |  |  |  |  |  |  |  |  |  |  |  |  |  |  |  |  |  |  |  |  |  |  |  |  | 1.00 | 0.90 | 0.42 | -0.25 | -0.02 | 0.20 | 0.46 | 0.12 | **TSS** |
|  |  |  |  |  |  |  |  |  |  |  |  |  |  |  |  |  |  |  |  |  |  |  |  |  |  |  | 1.00 | 0.53 | 0.13 | -0.13 | 0.33 | 0.38 | 0.05 | **Turb** |
|  |  |  |  |  |  |  |  |  |  |  |  |  |  |  |  |  |  |  |  |  |  |  |  |  |  |  |  | 1.00 | 0.20 | -0.87 | 0.20 | -0.12 | -0.73 | **Cond** |
|  |  |  |  |  |  |  |  |  |  |  |  |  |  |  |  |  |  |  |  |  |  |  |  |  |  |  |  |  | 1.00 | -0.10 | 0.03 | -0.15 | -0.32 | **pH** |
|  |  |  |  |  |  |  |  |  |  |  |  |  |  |  |  |  |  |  |  |  |  |  |  |  |  |  |  |  |  | 1.00 | 0.01 | 0.45 | 0.93 | **Depth** |
|  |  |  |  |  |  |  |  |  |  |  |  |  |  |  |  |  |  |  |  |  |  |  |  |  |  |  |  |  |  |  | 1.00 | -0.13 | 0.14 | **ADP** |
|  |  |  |  |  |  |  |  |  |  |  |  |  |  |  |  |  |  |  |  |  |  |  |  |  |  |  |  |  |  |  |  | 1.00 | 0.63 | **I_mean_** |
|  |  |  |  |  |  |  |  |  |  |  |  |  |  |  |  |  |  |  |  |  |  |  |  |  |  |  |  |  |  |  |  |  | 1.00 | **I_peak_** |
| * One of the EMC values of DahA (rain event C), which returned misleading correlation coefficients, was excluded from the data set as a suspicious outlier. No extra water was available to reanalyze the sample. | | | | | | | | | | | | | | | | | | | | | | | | | | | | | | | | | | |

## References:

IARC Working Group. (2010). Some non-heterocyclic polycyclic aromatic hydrocarbons and some related exposures. *IARC Monographs on the Evaluation of Carcinogenic Risks to Humans*, *92*. https://www.cabdirect.org/cabdirect/abstract/20113201374

Joa, K., Panova, E., Irha, N., Teinemaa, E., Lintelmann, J., & Kirso, U. (2009). *Determination of polycyclic aromatic hydrocarbons (PAHs) in oil shale processing wastes: Current practice and new trends*. https://doi.org/10.3176/OIL.2009.1.07

Khodadoust, A. P., Lei, L., Antia, J. E., Bagchi, R., Suidan, M. T., & Tabak, H. H. (2005). Adsorption of Polycyclic Aromatic Hydrocarbons in Aged Harbor Sediments. *Journal of Environmental Engineering*, *131*(3), 403–409. https://doi.org/10.1061/(ASCE)0733-9372(2005)131:3(403)

Monaco, D., Chianese, E., Riccio, A., Delgado-Sanchez, A., & Lacorte, S. (2017). Spatial distribution of heavy hydrocarbons, PAHs and metals in polluted areas. The case of “Galicia”, Spain. *Marine Pollution Bulletin*, *121*(1), 230–237. https://doi.org/10.1016/j.marpolbul.2017.06.003

Reed, D. A., & Stemer, T. R. (2002). *Total Petroleum Hydrocarbon Criteria Working Group (TPHCWG) Field Demonstration Report: Air Force Number 6 Fuel Farm, Dobbins AFB, GA* (AFRL-HE-WP-TR-2002-0158; p. 84). OPERATIONAL TECHNOLOGIES. https://apps.dtic.mil/sti/pdfs/ADA425422.pdf
